# Single-Nucleus RNA Sequencing and Spatial Transcriptomics Reveal Cellular Heterogeneity and Intercellular Communication Networks in the Hypothalamus–Pituitary–Ovarian Axis of Pregnant Mongolian Cattle

**DOI:** 10.3390/ani15152277

**Published:** 2025-08-04

**Authors:** Yanchun Bao, Fengying Ma, Chenxi Huo, Hongxia Jia, Yunhan Li, Xiaoyi Yang, Jiajing Liu, Pengbo Gu, Caixia Shi, Mingjuan Gu, Lin Zhu, Yu Wang, Bin Liu, Risu Na, Wenguang Zhang

**Affiliations:** 1 College of Animal Science, Inner Mongolia Agricultural University, Hohhot 010018, China; 2 Inner Mongolia Engineering Research Center of Genomic Big Data for Agriculture, Inner Mongolia Agricultural University, Hohhot 010018, China; 3 College of Veterinary Medicine, Inner Mongolia Agricultural University, Hohhot 010018, China; 4 Inner Mongolia Ben Niu Technology Co., Ltd., Hohhot 010018, China; 5 College of Life Science, Inner Mongolia Agricultural University, Hohhot 010018, China

**Keywords:** single-nucleus RNA sequencing, spatial transcriptomics, hypothalamic–pituitary–ovarian axis, cattle, reproductive regulation

## Abstract

The hypothalamus–pituitary–ovarian (HPO) axis orchestrates reproductive functions through intricate neuroendocrine crosstalk. Here, we integrated single-nucleus RNA sequencing (snRNA-seq) and spatial transcriptomics (ST) to decode the cellular heterogeneity and intercellular communication networks in the reproductive systems of pregnant Mongolian cattle. We retained a total of 6161 high-quality nuclei from the hypothalamus, 14,715 nuclei from the pituitary, and 26,072 nuclei from the ovary, providing a comprehensive cellular atlas across the HPO axis. In the hypothalamus, neurons exhibited synaptic and neuroendocrine specialization, with glutamatergic subtype Glut4 serving as a TGFβ signaling hub to regulate pituitary feedback, while GABAergic GABA1 dominated PRL signaling, likely adapting maternal behavior. Pituitary stem cells dynamically replenished endocrine populations via TGFβ, and lactotrophs formed a *PRL*–*PRLR* paracrine network with stem cells, synergizing mammary development. Ovarian luteal cells exhibited steroidogenic specialization and microenvironmental synergy: endothelial cells coregulated TGFβ-driven angiogenesis and immune tolerance, while luteal–stromal *PRL*–*PRLR* interactions amplified progesterone synthesis and nutrient support. Granulosa cells (GCs) displayed spatial-functional stratification, with steroidogenic GCs persisting across pseudotime as luteinization precursors, while atretic GCs underwent apoptosis. Spatial mapping revealed GCs’ annular follicular distribution, mediating oocyte–somatic crosstalk, and luteal–endothelial colocalization supporting vascularization. This study unveils pregnancy-specific HPO axis regulation, emphasizing multi-organ crosstalk through TGFβ/PRL pathways and stem cell-driven plasticity, offering insights into reproductive homeostasis and pathologies.

## Simple Summary

The hypothalamus–pituitary–ovarian (HPO) axis regulates reproduction, yet its pregnancy-specific adaptations remain unclear. Here, we integrated single-nucleus RNA sequencing and spatial transcriptomics to profile the reproductive organs of pregnant Mongolian cattle. We found that hypothalamic neurons mediated TGFβ and prolactin signaling, while pituitary stem cells supported endocrine renewal and lactotrophs formed PRL–PRLR networks promoting mammary development. In the ovary, luteal and endothelial cells co-regulated angiogenesis and progesterone synthesis, and granulosa cells showed spatial-functional stratification and follicular localization. These findings reveal pregnancy-adapted HPO regulation via TGFβ/PRL pathways and stem cell plasticity.

## 1. Introduction

Mongolian cattle (*Bos taurus*) are a native breed found in the arid and cold regions of the Mongolian Plateau. They possess strong cold resistance and drought tolerance [1,2,3]. For a long time, they have provided local herders with meat, dairy products, and draft power. Despite their adaptability and moderate productivity under harsh environmental conditions, their reproductive efficiency remains low. Issues such as reduced conception rates and high early embryonic loss continue to limit genetic improvement and sustainable development [4,5]. Research into the physiological basis of these reproductive challenges has increasingly focused on the hypothalamic–pituitary–ovarian (HPO) axis, which plays a central role in regulating reproductive hormones [6]. The dysfunction in the HPO axis, particularly insufficient progesterone (P4) secretion or abnormal gonadotropin-releasing hormone (GnRH) signaling, can lead to embryo implantation failure or pregnancy loss [7,8]. However, the precise molecular mechanisms governing the HPO axis during pregnancy in Mongolian cattle remain unclear. Therefore, systematically deciphering the molecular mechanisms controlling the HPO axis during pregnancy is essential for improving reproductive management and conserving this genetically valuable breed.

During pregnancy, the HPO axis undergoes significant changes to maintain the endocrine environment required for fetal development. Elevated progesterone and estrogen (E2) levels exert negative feedback on the hypothalamus, reducing GnRH pulse frequency and amplitude, thereby suppressing follicular recruitment and ovulation [9,10]. This leads to decreased secretion of follicle-stimulating hormone (FSH) and luteinizing hormone (LH) by the anterior pituitary, preventing new dominant follicle formation. Concurrently, pituitary-derived prolactin (PRL) increases substantially, facilitating mammary gland development and lactation preparation [11]. The ovary becomes relatively quiescent. Initially, the corpus luteum secretes P4 and E2 before the placenta assumes predominant endocrine functions [12]. This coordinated regulation of the HPO axis maintains hormonal homeostasis critical for pregnancy maintenance.

Cells with a diameter of more than 50 μm in the HPO axis present considerable challenges for single-cell RNA sequencing (scRNA-seq) [13]. Single-nucleus RNA sequencing (snRNA-seq) overcomes these barriers by profiling nuclear transcripts, enabling high-resolution analysis, even in archived or dense tissues. Complementary spatial transcriptomics (ST) technologies further allow in situ mapping of gene expression, revealing spatial cellular interactions. While extensive single-cell studies in other species have elucidated cellular heterogeneity and signaling networks within the HPO axis [14,15,16,17,18], systematic characterization of these features during pregnancy in Mongolian cattle remains lacking. This knowledge gap restricts understanding of the molecular adaptations enabling this breed to maintain pregnancy under extreme environmental conditions.

Despite significant progress in reproductive studies across domestic animals, our understanding of the cellular diversity and spatial organization of the HPO axis during pregnancy in Mongolian cattle remains limited. Given their unique reproductive resilience under extreme environmental conditions, Mongolian cattle may possess distinct cellular and molecular mechanisms that regulate gestation. Key signaling pathways, such as TGFβ and PRL, are likely to play critical roles in coordinating hormonal crosstalk among the hypothalamus, pituitary, and ovary. To address this gap, this study will apply snRNA-seq and spatial transcriptomics (ST) to profile the transcriptional states and spatial distribution of HPO cells during pregnancy. By elucidating these mechanisms, we aim to provide new insights into reproductive adaptation and offer a theoretical basis for improving fertility, enhancing embryo survival, and developing targeted reproductive management strategies in Mongolian cattle raised in harsh environment.

## 2. Materials and Methods

### 2.1. Tissue Dissociation and Preparation

In November 2023, the experiment was conducted in Alxa Left Banner, Alxa League, Inner Mongolia Autonomous Region, focusing on the dissection and tissue sampling of a five-year-old, clinically healthy pregnant Mongolian cow, which was sourced from a local herder’s pasture. Prior to slaughter, the cow underwent a veterinary ultrasound examination to confirm pregnancy status, and fetal crown–rump length was measured postmortem to further determine gestational age. Based on these measurements, the cow was confirmed to be in mid-gestation. Although ultrasound images were not retained, pregnancy was verified by experienced veterinarians. To minimize biological variability and focus on pregnancy-specific transcriptional regulation, only this individual was used for tissue sampling, with multiple technical replicates processed per tissue type.

Following slaughter, fresh hypothalamic, pituitary, and bilateral ovarian tissues were immediately harvested. The two ovaries were processed separately: the left ovary was designated for snRNA-seq, while the right ovary was preserved for ST. All maternal tissues were first rinsed with physiological saline and then washed with ice-cold 1× phosphate-buffered saline (PBS, HyClone, Logan, UT, USA) to remove residual blood and connective tissue. After blotting with absorbent paper, tissues were dissected on dry ice. The hypothalamus and pituitary were cut into approximately 3 mm cubes, while the ovary was sampled to include key functional structures such as the corpus luteum, small follicles, antral follicles, and dominant follicles. Tissue fragments were snap-frozen in liquid nitrogen for at least 2 min and stored long-term in cryovials to preserve RNA integrity and cellular architecture.

### 2.2. Nuclei Isolation and Library Preparation

Three trimmed tissue samples (hypothalamus, pituitary, and ovary) were processed using a Singulator 100 instrument (S2 Genomics, Livermore, CA, USA) with 2 mL of lysis buffer (LB) per sample, following the standard single-nucleus preparation protocol. Nuclei were isolated using a Tissue Nuclei Extraction Kit (Biotechnology, Shanghai, China). All buffers used during isolation, including LB, Nuclei Buffer (NB), and Nuclei Buffer with RNA protection (NB-RNA), were supplemented with 1% bovine serum albumin (BSA; Solarbio, Beijing, China) and RNase inhibitor at a final concentration of 2 U/μL (Vazyme, Vazyme, Nanjing, China). All procedures were performed under RNase-free conditions using low-retention, sterile consumables, and pre-chilled solutions. The nuclear suspension was collected after Singulator processing, centrifuged at 500× *g* for 5 min at 4 °C, and resuspended in ice-cold LB containing BSA. The suspension was then layered onto PB2/PB3 solutions and centrifuged at 3000× *g* for 20 min at 4 °C to isolate nuclei from the interface. The purified nuclei were washed, filtered through a 40 μm sterile cell strainer, and resuspended in NB-RNA. Nuclear quality was assessed using the Countstar FL automated fluorescence cell counter (Countstar Biotech, Shanghai, China), ensuring the following criteria: viability ≥90%, aggregation rate <5%, nuclear purity >70%, concentration between 300–2000 nuclei/μL, and size range 5–30 μm. Contamination control was performed through strict RNase-free handling, visual inspection of nuclear suspensions, and inclusion of blank controls during downstream steps. Qualified nuclei were then combined with barcoded gel beads and enzyme mixtures on a 10× Genomics Chromium Controller (10× Genomics, Pleasanton, CA, USA) to generate gel beads-in-emulsions (GEMs). Within each GEM, nuclei lysis and reverse transcription occurred, incorporating 10× barcodes and UMIs into cDNA molecules. After emulsions were broken, cDNA was purified and amplified via PCR. The amplified cDNA was then used for library construction, including fragmentation, end repair, A-tailing, adapter ligation, and sample index PCR amplification. Sequencing libraries were quality-checked for fragment size distribution and concentration. Final libraries were size-selected and sequenced on an Illumina NovaSeq 6000 platform using the S4 flow cell and a 300-cycle reagent kit (Illumina, San Diego, CA, USA), following the standard 10× Genomics protocol for snRNA-seq.

### 2.3. Single Nucleus RNA Sequencing Data Processing and Analysis

After sequencing, the mkfastq function within Cell Ranger software (version 7.1.0) employing the Illumina’s bcl2fastq tool was used to demultiplex the raw sequencing data (BCL files) [19]. This demultiplexing process utilizes cell-specific barcodes and unique molecular identifiers (UMIs) embedded within the sequencing reads to separate them into individual samples or cells. The sequencing reads are then compiled into a FASTQ file, including both the sequence reads and their corresponding quality scores. Subsequently, these reads were aligned to the *Bos taurus* genome (ARS-UCD1.2) using the STAR (version 2.4.2a) aligner to generate count matrices [20]. During the count step, Cell Ranger performed stringent filtering of raw reads to ensure high-quality data. Reads with low base quality scores, unrecognized barcodes, or invalid UMIs were discarded. Only reads with barcodes matching the 10× Genomics whitelist—allowing for a single-base mismatch correction—were retained. Read2 sequences that failed to align confidently to the reference genome or mapped ambiguously were excluded from downstream analysis. UMI duplicates were removed by collapsing identical UMIs that mapped to the same gene and cell. In addition, cell barcodes were identified based on UMI counts using a knee-plot algorithm, with the expect-cells = 1000 parameter guiding the cell-calling threshold. All other parameters were used as Cell Ranger v7.1.0 defaults, including hemistry = auto, localcores = 8, and localmem = 64. Background ambient RNA and empty droplets were filtered out automatically, ensuring that only true high-confidence nuclei were included in the final gene expression matrix. These matrices and associated metadata were imported into the Seurat package (version 5.1.0) in R for further analysis [21]. To filter out low-complexity, doublet, or apoptotic cells, cells with expressed genes ranging between 200 and 4000 and a mitochondrial gene content below 10% of the total genes were retained for further analysis. The remaining data were normalized using the NormalizeData function with default parameters: normalization.method = “LogNormalize” and scale.factor = 10,000. Subsequently, the top 2000 highly variable genes were identified using the FindVariableFeatures function with selection.method = “vst” and nfeatures = 2000. The variable genes were centered and scaled to unit variance using the ScaleData function. Dimensionality reduction was then performed on the top 20 principal components using the RunPCA function. The number of significant principal components was evaluated using the JackStraw and JackStrawPlot functions. To account for potential batch effects and technical variability across ovarian subregions (corpus luteum, small follicles, antral follicles, and dominant follicles), data integration was performed using Seurat’s built-in CCA-based integration framework, followed by mutual nearest neighbor (MNN) alignment. Additionally, RunHarmony() was used with default parameters to further correct residual batch effects. UMAP dimensional reduction was performed using RunUMAP (dims = 1:20).

### 2.4. Cell Clustering and Cell Type Identification

Cell clustering was conducted using the FindNeighbors and FindClusters functions with the Louvain algorithm. The relationships between clusters were visualized across a resolution parameter range of 0.4 to 1.2 using the clustree package (version 0.5.1) [22]. Optimal resolutions for the hypothalamus, pituitary, and ovary were determined to be 0.8, 0.8, and 0.6. Cell clusters were annotated based on canonical markers reported in the literature. Differentially expressed genes (DEGs) in the hypothalamus, pituitary, and ovary of Mongolian cattle were identified using the FindAllMarkers function (Wilcoxon rank sum test), with parameters: min.pct = 0.25, logfc.threshold = 0.25, and adjusted *p*-value < 0.05. Finally, Seurat generated visualizations including DoHeatmap, VlnPlot, FeaturePlot, and DotPlot to illustrate the results. Functional enrichment analysis was performed using the clusterProfiler package (v.4.12.2) from Bioconductor in R, with the org.Bt.eg.db database [23]. Gene Ontology (GO) and Kyoto Encyclopedia of Genes and Genomes (KEGG) functional enrichment were conducted using the enrichGO and enrichKEGG functions with default *p*-value cutoff (0.05) and p.adjust method (“BH”). Visualizations were generated with the ggplot2 package (v.3.5.1) [24].

### 2.5. High-Dimensional Weighted Gene Co-Expression Network Analysis

High-dimensional weighted gene co-expression network analysis (hdWGCNA) was implemented in R to construct co-expression networks across multiple cellular and spatial dimensions, thereby revealing complex gene relationships [25]. The process begins by selecting genes expressed in at least 5% of cells to construct the hdWGCNA object, ensuring that only genes with sufficient expression levels are included. Using the MetacellsByGroups function, a metacell gene expression matrix was built, which aggregates cells with similar expression profiles to reduce dimensionality while preserving biological relevance. To determine an appropriate threshold for constructing the co-expression network, the TestSoftPowers function was applied with the network type set to ‘signed’, scanning across various soft power thresholds to identify the optimal value that ensures a robust gene–gene adjacency matrix by eliminating weak connections. Once the optimal soft threshold was determined, the ConstructNetwork function was used with setDatExpr set to FALSE to build the co-expression network, generating a weighted adjacency matrix that reflects gene co-expression relationships. Module eigengenes (MEs), which summarize the expression profiles of entire modules, were then calculated using the ModuleEigengenes function, which performs PCA on subsets of the gene expression matrix for each module. To assess the importance of modules, the GetModuleTraitCorrelation function was employed to measure correlations between modules and traits, evaluating significance based on *p*-values and correlation coefficients. Finally, the PlotModuleTraitCorrelation function was used to visualize these correlations through heatmaps, clearly representing module-trait associations.

### 2.6. Pseudotime Analysis

To infer developmental trajectories, pseudotime analysis was conducted using Monocle 2 [26]. A Monocle object was created from Seurat data using the newCellDataSet function with lowerDetectionLimit = 0.5. Data normalization used estimateSizeFactors and estimateDispersion (default). Dimensionality reduction was performed using reduceDimension (method = “DDRTree”), and trajectories were constructed using orderCells with default branch/root parameters. Gene expression trends were visualized using plot_genes_in_pseudotime and plot_pseudotime_heatmap with the top variable genes. To evaluate the differentiation potential of gonadotroph subtypes, we applied Cyto-TRACE (version 0.3.3), a computational tool designed to infer cellular plasticity and developmental state from single-cell transcriptomic data. The analysis was performed using normalized expression data from Seurat, which were converted into a gene-by-cell matrix compatible with CytoTRACE using the as.matrix function in R (version 4.4.1). CytoTRACE was run with default parameters, which rank each cell based on the number of expressed genes and gene diversity to infer differentiation potential. The resulting CytoTRACE scores range from high to low, providing a pseudotime-like trajectory of cellular maturation. Visualization of differentiation states across gonadotroph clusters was performed by integrating CytoTRACE scores with UMAP embeddings from the Seurat object. Genes positively or negatively correlated with differentiation states were extracted using the getCytoTRACE and cor functions, and the top correlated genes were further investigated for their known roles in pituitary development and hormone regulation.

### 2.7. Cell-Cell Communication Analysis

To comprehensively understand intercellular interactions, the CellChat v.1.6.1 package in R was employed to infer and analyze cell communication [27]. CellChatDB.human was used as the reference ligand–receptor database, and bovine homologs were mapped accordingly. Using the CellChat and patchwork packages (version 1.3.0), a CellChat object was created to establish a comprehensive database of ligand–receptor interactions. Preprocessing of expression data was performed to facilitate cell communication analysis. Default pipeline functions—including identifyOverExpressedGenes, identifyOverExpressedInteractions, computeCommunProb, and computeCommunProbPathway—were used unless otherwise stated. Circular plots were utilized to visually illustrate signaling pathways enriched between different cell populations, emphasizing the modular nature of these interactions. The contribution of each ligand–receptor pair to these signaling pathways was calculated and visualized, offering insights into the role of individual ligand–receptor pairs in regulating cell communication. Finally, CellChat was used to infer biologically meaningful communication patterns and a systematic analysis of the cell communication network was conducted to identify key signaling roles within cell populations, such as primary senders and receivers, as well as their significant contributions to specific signaling pathways.

### 2.8. Stereo-Seq Tissue Preparation and Sequencing

Prior to initiating the experiment, sufficient dry ice was prepared and ground into crushed ice, while OCT embedding medium, lint-free absorbent paper, and plastic embedding cassettes were pre-cooled. Directional markers were labeled on the cassettes using a permanent marker. Pre-cooled OCT medium was applied from the center to the periphery at the base of each cassette to minimize bubble formation. Freshly resected right ovarian tissues were trimmed to dimensions not exceeding the Stereo-seq GE chip area (1.1 cm × 1.1 cm), with surfaces gently blotted using lint-free paper to remove residual liquid before proceeding to fixation, hematoxylin–eosin (H&E) staining, permeabilization, and in situ reverse transcription. To capture comprehensive spatial information, ovarian tissues were sectioned from two different anatomical orientations and processed separately for Stereo-seq spatial transcriptomic profiling. Amplified cDNA was subsequently used for library preparation and sequenced on the DNBSEQ-T7 platform. Bioinformatics analysis was conducted in Python (version 3.7). To spatially map the cell types that we annotated in snRNA-seq data to spatial transcriptomic data, we applied cell2location (version 0.7.0) to integrating snRNA-seq data with Stereo mRNA count matrices, as described previously [28]. Cell2location maps fine-grained cell types in spatial transcriptomics. In brief, the cell2location model estimates the abundance of each cell population in each location by decomposing mRNA counts in Stereo data using the transcriptional signatures of reference cell types. Following cell type annotation of integrated Scanpy (version 1.8.0) objects, reference signatures were computed via negative binomial regression using default parameters in Cell2location. The max_epochs parameter was optimized by monitoring ELBO loss trajectories until stabilization. The following parameters were used to train the model: ‘max_epochs’ = 1000, ‘batch_size’ = 2000, ‘train_size’ = 1 and ‘Ir’ = 0.002. For spatial mapping, the number of cells per location N = 30 and ‘detection_alpha’= 20, followed by spatial visualization using sc.pl.spatial functions. Spatially co-occurring cell types were identified through non-negative matrix factorization (NMF) with default parameters to delineate cellular compartments.

## 3. Results

### 3.1. Identification of Cell Types in Three Tissues of Mongolian Cattle HPO Axis Using Single Nucleus Transcriptomes

**Figure 1:**
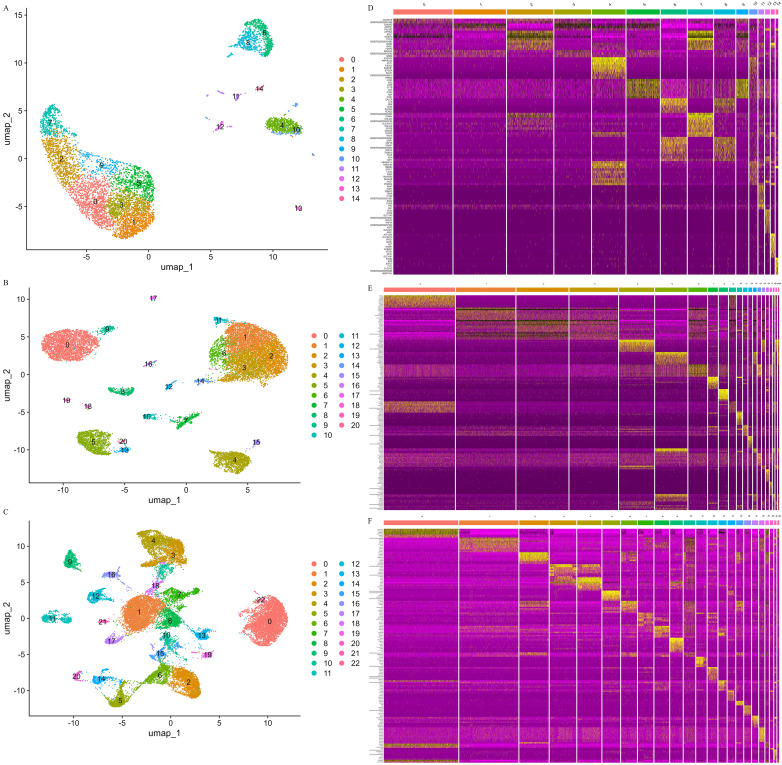
Clustering and differentially expressed genes of Mongolian cattle HPO axis cells. (**A**–**C**): UMAP visualization of all cells in the hypothalamus, pituitary and ovary. (**D**–**F**): Heatmap showing the top 10 DEGs in the hypothalamus, pituitary, and ovary.

To investigate the HPO axis during pregnancy, we performed a comprehensive snRNA-seq analysis using Mongolian cattle as our model organism. Tissue samples were systematically collected from the hypothalamus, pituitary, and ovary, followed by high-resolution snRNA-seq to generate robust transcriptomic profiles. During the initial data preprocessing phase, we implemented stringent quality control protocols to ensure data integrity and reliability (Supplementary Figure S1A). Nuclei were filtered based on three key parameters: the number of expressed genes, total molecular counts per cell, and the proportion of mitochondrial genes. This rigorous quality control process effectively eliminated low-quality nuclei, doublets, and apoptotic cells, resulting in the retention of high-quality nuclei for subsequent analyses (Supplementary Figure S1B–D). Following quality control, we obtained 6161 high-quality nuclei from the hypothalamus, 14,715 from the pituitary, and 26,072 from the ovary. To characterize the cellular heterogeneity within these tissues, we employed UMAP for dimensionality reduction and visualization of cellular similarities. This analysis revealed distinct cellular clusters across the three tissues: 15 in the hypothalamus (Figure 1A), 21 in the pituitary (Figure 1B), and 23 in the ovary (Figure 1C). Each cluster exhibited unique transcriptional signatures, suggesting specialized functional roles within the HPO axis during pregnancy (Figure 1D–F).

**Figure 2:**
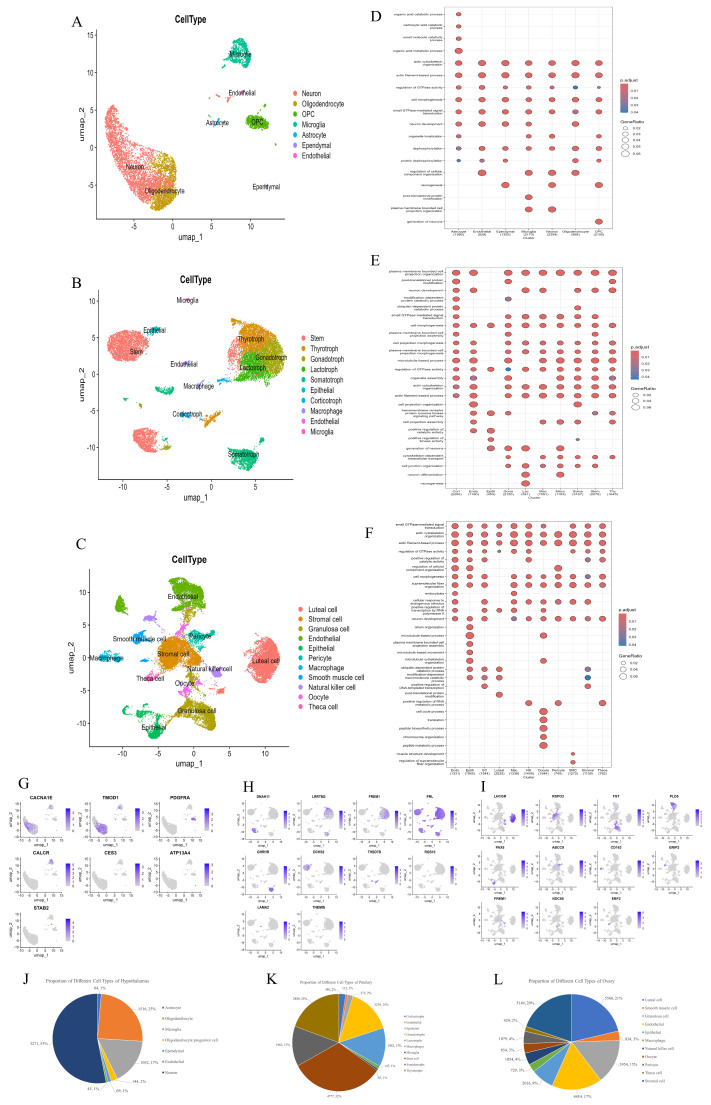
Identification of cell types across all HPO axis tissues. (**A**–**C**): UMAP plots showing various cell types in the hypothalamus, pituitary and ovary. (**D**–**F**): GO enrichment of DEGs in various cell types in hypothalamus, pituitary, and ovarian tissues. (**G**–**I**): UMAP analysis revealing gene expression heterogeneity among cell populations in the hypothalamus, pituitary, and ovary. (**J**–**L**): The proportion of each cell type in the hypothalamus, pituitary, and ovary.

In the hypothalamus, 15 transcriptionally distinct clusters (C0–C14) were classified into seven major cell types (Figure 2A and Figure S2A). To better understand the functional specialization of each cell type in the hypothalamus, we performed GO enrichment analysis based on DEGs identified across the seven major cell types (Figure 2D). Neurons showed enrichment in neurogenesis, synaptic signaling, and cytoskeletal organization, consistent with their roles in neuroendocrine regulation. Astrocytes were associated with metabolic processes and actin filament-based organization, highlighting their supportive functions. Microglia were enriched in protein dephosphorylation and cellular component organization, reflecting immune surveillance and synaptic remodeling. Oligodendrocytes and OPCs showed enrichment in cell projection organization and neurogenesis, suggesting roles in myelination and precursor differentiation. Ependymal cells were linked to morphogenesis and neuron development, while endothelial cells were associated with protein modification and barrier-related functions. These findings underscore the coordinated functional diversity of hypothalamic cell types during mid-gestation. Neuron cells (C0, C2, C3, C7, C9 and C11), characterized by high expression of *CACNA1E*—a voltage-gated calcium channel gene essential for synaptic transmission—accounted for 53% of hypothalamic cells in our study (Figure 2J). Non-neuronal populations included oligodendrocytes (C1 and C5; high expression of *TMOD1*), oligodendrocyte progenitor cells (C4 and C10; high expression of *PDGFRA*), microglia (C6 and C8; high expression of *CALCR*), astrocytes (C12; high expression of *CES3*), ependymal cells (C13; high expression of *ATP13A4*), and endothelial cells (C14; high expression of *STAB2*), collectively highlighting the hypothalamus’s dual role in neuro–glial crosstalk and microenvironmental homeostasis (Figure 2G and Figure S2D).

Within the pituitary, 10 cell types were identified (Figure 2B and Figure S2B), including five endocrine lineages: lactotrophs (C3; high expression of *PRL*), corticotrophs (C10 and C14; high expression of *THSD7B*), somatotrophs (C4, C8, C15, C19 and C20; high expression of *GHRHR*), gonadotrophs (C2, C13 and C18; high expression of *FREM1*), and thyrotrophs (C1, C7 and C11; high expression of *LRRTM3*) (Figure 2G and Figure S2E). Stem cells (C0, C5 and C6; high expression of *DNAH11*) dominated the pituitary (32% of total cells), suggesting dynamic endocrine cell replenishment (Figure 2K). Non-endocrine populations—macrophages (C12; high expression of *RGS10*), microglia (C17; high expression of *THEMIS*), and epithelial cells (C9; high expression of *DCHS2*)—likely contribute to immune and structural maintenance. GO enrichment analysis revealed cell type-specific functional programs (Figure 2E). Lactotrophs were enriched in cell projection morphogenesis and junction organization, reflecting structural plasticity and secretory activity. Corticotrophs and somatotrophs showed enrichment in post-translational modification and ubiquitin-related processes, supporting roles in peptide hormone synthesis and regulation. Gonadotrophs and thyrotrophs were associated with actin cytoskeleton organization, GTPase signaling, and transmembrane receptor pathways, indicating responsiveness to hypothalamic input and involvement in reproductive and metabolic control. Stem cells were enriched in microtubule-based processes, organelle assembly, and morphogenesis, consistent with their active proliferative and differentiation potential. Macrophages and microglia showed enrichment in kinase activity regulation and cellular component organization, suggesting roles in immune surveillance and microenvironment modulation. Epithelial cells were associated with cell projection organization, actin filament-based processes, and junction regulation, in line with their barrier and support functions. These enrichment patterns collectively highlight the distinct functional specializations of pituitary cell types, integrating hormone production, structural remodeling, regeneration, and immune regulation during pregnancy.

In the ovary, 23 clusters were annotated into 11 cell types (Figure 2C and Figure S2C). Luteal cells (C0, C19 and C22; high expression of *HSD3B1*), critical for progesterone synthesis, and GCs (C2, C6 and C10; high expression of *FST*), key regulators of folliculogenesis, comprised 21% and 15% of ovarian cells, respectively (Figure 2L). Stromal components included smooth muscle cells (C12 and C21; high expression of *GRIP2*), endothelial cells (C3, C4 and C9; high expression of *PLD5*), macrophages (C11; high expression of *CD163*), oocytes (C15 and C18; high expression of *NDC80*), pericytes (C7; high expression of *ABCC9*) and theca cells (C17; high expression of *EBF2*). Notably, the key cell population (stromal cells, which regulate pregnancy maintenance) was also identified (C1 and C8; high expression of *RSPO3*) (Figure 2I and Figure S2F). GO enrichment analysis showed the biological function of each cell type (Figure 2F). Luteal cells were enriched in GTPase signaling and morphogenesis, reflecting their roles in hormone secretion and structural remodeling. Granulosa cells showed enrichment in cytoskeletal organization and transcriptional regulation, supporting their involvement in follicle growth and endocrine signaling. Oocytes were associated with RNA metabolism and chromosome organization, consistent with meiotic and developmental competence. Stromal cells were linked to microtubule-based processes and peptide biosynthesis, indicating structural and paracrine support during pregnancy. Endothelial cells and pericytes showed enrichment in cytoskeletal and membrane projection pathways, highlighting roles in angiogenesis and vascular stability. Macrophages were enriched in protein catabolism and cell cycle regulation, suggesting functions in immune surveillance and tissue homeostasis. Theca cells exhibited signal transduction and cytoskeletal remodeling functions, which are consistent with steroidogenesis. SMCs were associated with actin-based organization, reflecting contractile activity. NK cells were enriched in transcriptional regulation and cellular organization, indicating immune activation roles. These patterns underscore the coordinated specialization of ovarian cells in steroidogenesis, immune regulation, tissue remodeling, and follicular support during pregnancy.

### 3.2. Differentiation Trends of Neuronal Subtypes and Cell–Cell Communication in the Hypothalamus

To elucidate the molecular heterogeneity and interaction mechanisms of hypothalamic neuronal subtypes involved in the regulation of the HPO axis, we conducted a comprehensive series of analyses. Initially, based on the expression of glutaminase (*GLS*) and gamma-aminobutyric acid type B receptor subunit 2 (*GABBR2*), we refined the clustering of neuronal populations, successfully identifying seven glutamatergic neuronal subtypes (Glut1–Glut7) and one GABAergic neuronal subtype (GABA1) (Supplementary Figure S3A,B). Subsequently, to unravel the intricate regulatory mechanisms among these neuronal subtypes, we performed a predictive analysis of cell–cell communication. Among the eight neuronal clusters, we identified 102 ligand–receptor pairs, which were further classified into 31 signaling pathways. Of these, the transforming growth factor-beta (TGFβ), prolactin (PRL), and FSH signaling pathways were prioritized for their critical roles in reproductive regulation.

**Figure 3:**
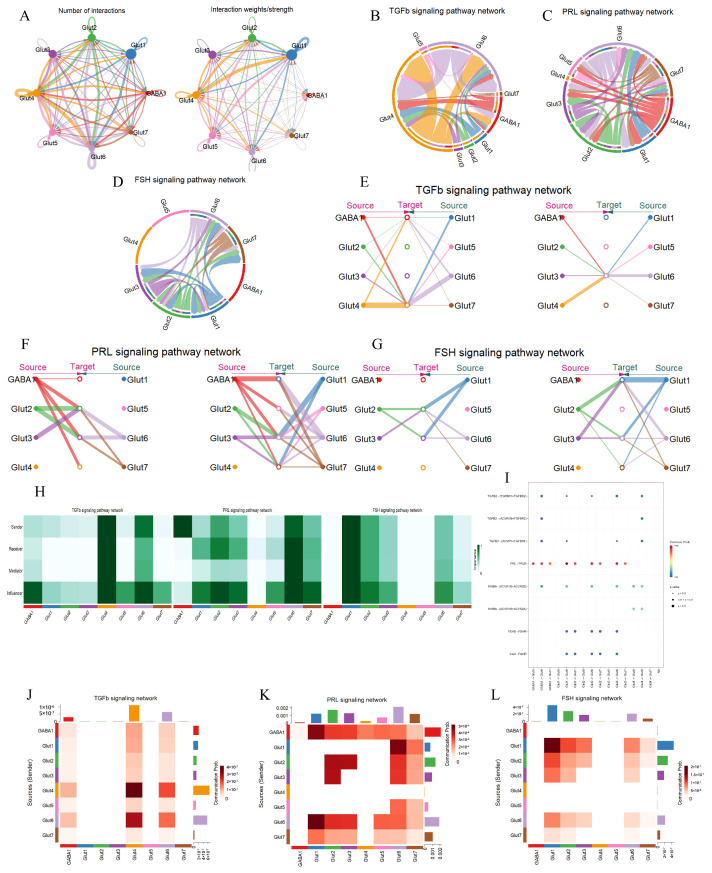
Signaling pathway networks and cellular communication of neuronal subtypes in the hypothalamus. (**A**): Circle plot illustrating the number of interactions between the various cell types and their weight. The arrows point from the ligand-expressing cells to the receptor-expressing cells, the size of the circles indicates the number of cells, and the width of the lines indicates the number of ligand–receptor pairs. (**B**–**D**): Chordplots showing the direction and strength of interactions of TGFβ, FSH, and PRL signaling pathways between different cell types in the hypothalamus. (**E**–**G**): Hierarchical plots showing the intercellular communication network of TGFβ, PRL and FSH signaling pathways. The size of the circle indicates the number of cells, and the width of the line indicates the communication probability. (**H**): Heatmap showing the relative importance of cell type in TGFβ, FSH, and PRL signaling pathways. Mediator is a compute unit that controls the communication flow between any two compute unit groups. Influencer is a cell that controls the information flow in a signaling network. Importance is the likelihood of the cell type playing the four roles (sender, receiver, mediator, and influencer). A darker color indicates that the cell is playing a bigger role. (**I**): Bubble plot showing the strength of the interaction of ligand–receptor pairs in cellular communication in the TGFβ, FSH, and PRL signaling pathways. (**J**–**L**): Heatmaps showing the intensity of cellular communication in the TGFβ, FSH, and PRL signaling pathways.

In the analysis of cellular interactions, Glut4 emerged as the most active cluster, exhibiting the highest number and weight of signaling interactions (Figure 3A). This prominence is likely attributed to its dominant role in the TGFβ signaling pathway (Figure 3B). Network centrality analysis identified Glut4 and Glut6 as the primary ligand sources for TGFβ signaling (Figure 3E). Additionally, Glut4, Glut6, and GABA1 clusters expressed *TGFBR1* and *ACVR1* receptor genes (Figure 3I), which are closely associated with negative feedback regulation between the hypothalamus and pituitary, indicating their roles as key targets in the TGFβ pathway. Role significance analysis revealed that Glut4 plays the most critical role, while Glut6 functions as both a sender and an influencer, demonstrating its capacity for autocrine and paracrine signaling. In contrast, GABA1, despite high expression of ligand genes, primarily acts as an influencer within this pathway (Figure 3H,J). Given the gestational status of the experimental cow, we identified the PRL signaling pathway within the hypothalamus (Figure 3C). In this pathway, all neuronal clusters except Glut4 exhibited high activity, suggesting a complex regulatory network (Figure 3F). Specifically, GABA1 was identified as the primary sender in the PRL pathway, with significant expression of *PRL* ligand genes (Figure 3I). Other clusters functioned as senders, receivers, mediators, and influencers, all expressing high levels of *PRLR*, indicating their dual capacity for autocrine and paracrine signaling (Figure 3H,K). In the FSH signaling pathway, no cell–cell communication was detected among GABA1, Glut4, and Glut5 clusters (Figure 3D,H,L). Network centrality and role significance analyses identified Glut1 as the primary ligand source and receptor target in this pathway (Figure 3G). However, no significant co-expression of ligand and receptor genes was observed among these neuronal subtypes (Figure 3I), suggesting that FSH-mediated regulation in the hypothalamus may rely on extrinsic inputs (e.g., pituitary-derived FSH) rather than intrinsic neuronal crosstalk. These findings underscore the complexity of inter-pathway communication within the hypothalamus, where pituitary and ovarian hormones exert feedback regulation to modulate the synthesis and release of various neuropeptides.

To further explore the differentiation relationships among neuronal subclusters, we performed trajectory analysis using the Monocle2 algorithm. The inferred trajectory revealed three distinct differentiation states: Glut4 represented the earliest neuronal state, followed sequentially by Glut5, Glut3, Glut7, and Glut2, with Glut1 positioned at a critical branching node, ultimately differentiating into GABA1 (Supplementary Figure S3C,D). Along this pseudotime timeline, the expression patterns of *ABCC13*, *ENSBTAG00000049814*, and *NLGN1* exhibited the most significant changes. Specifically, *ENSBTAG00000049814* displayed a biphasic expression pattern (initial decrease followed by an increase), *NLGN1* was upregulated in the terminal differentiation phase, and *ABCC13* showed an initial increase followed by a decline (Supplementary Figure S3E). These dynamic gene expression patterns provide critical insights into the molecular mechanisms underlying neuronal subtype differentiation. Our findings reveal a sophisticated signaling network within the hypothalamus, where TGFβ and PRL pathways dominate intraneuronal communication, while FSH signaling depends on extrinsic hormonal inputs. The identification of Glut4 as a regulatory hub and GABA1 as a PRL-centric influencer highlights the functional specialization of neuronal subtypes in gestational adaptation.

### 3.3. Cell–Cell Communication Among Various Cell Types in the Pituitary

**Figure 4:**
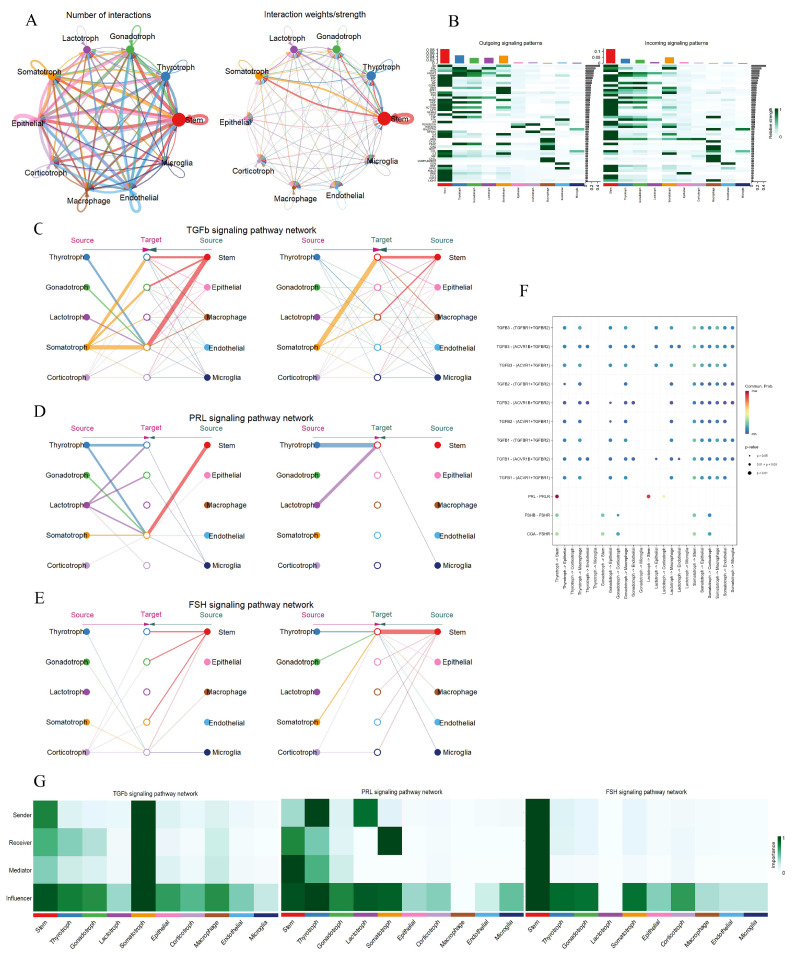
Signaling pathway networks and cell communication of various cell types within pituitary. (**A**): Circle plot illustrating the number of interactions between the various cell types and their weight. (**B**): Identified signaling pathways between all cell types; darker colors indicate a higher degree of cell involvement in the pathway. (**C**–**E**): Hierarchical plots showing the intercellular communication network of TGFβ, PRL and FSH signaling pathways. (**F**): Bubble plot showing the strength of the interaction of ligand–receptor pairs in cellular communication in the TGFβ, FSH, and PRL signaling pathways. (**G**): Heatmap showing the relative importance of cell type in TGFβ, FSH, and PRL signaling pathways.

During pregnancy, inter-cellular communication among diverse pituitary cell types is of paramount importance for the precise coordination of hormone secretion. This coordinated secretion is essential to meet the intricate requirements of fetal development and to uphold the normal physiological state of pregnancy. In this study, a comprehensive and systematic analysis of cell–cell communication within the pituitary was conducted. A total of five endocrine and five non-endocrine cell types were identified. Through analysis, 166 ligand–receptor pairs were recognized and further classified into 48 distinct signaling pathways within the pregnant pituitary. Notably, the TGFβ, PRL, and FSH signaling pathways, which were previously identified in the hypothalamus, were also detected in the pituitary. This discovery has spurred the need for further in-depth investigations. Cell interaction maps demonstrated that pituitary stem cells exhibit the highest frequency and intensity of interactions (Figure 4A). Following stem cells, thyrotrophs, gonadotrophs, lactotrophs, and somatotrophs showed decreasing levels of interaction activity (Figure 4B). Network centrality analysis of the TGFβ pathway indicated extensive cross-talk among different cell types (Figure 4C). Somatotrophs displayed robust autocrine capabilities, as evidenced by their expression of the receptor genes TGFBR1, TGFBR2, and ACVR1 (Figure 4F). They also engaged in paracrine signaling with stem cells. Gonadotrophs mainly participated in the TGFβ pathway through autocrine signaling, as supported by role-based analysis, while other cell types predominantly functioned as influencers (Figure 4G). In the PRL pathway, lactotrophs served as hub cells, primarily interacting with stem cells through paracrine signaling. Thyrotrophs promoted the expression of the PRL pathway via both autocrine and paracrine signaling with stem cells (Figure 4D). Ligand–receptor pair analysis revealed that the interaction between lactotrophs and stem cells, mediated by the PRL ligand and PRLR receptor genes, played a pivotal role (Figure 4F,G). In the FSH pathway, stem cells emerged as the most significant players. They interacted with five endocrine cell clusters through autocrine and paracrine signaling, thereby promoting the expression of the FSH pathway (Figure 4E). Stem cells were particularly notable for their expression of the CGA ligand gene (Figure 4F). Remarkably, stem cells occupied central positions in all three pregnancy-related signaling pathways. In the FSH pathway, they assumed four distinct functions: sender, receiver, mediator, and influencer, underscoring their substantial regulatory capabilities in pregnant Mongolian cattle. Although gonadotrophs had relatively weak interactions with other cell types, they mainly acted as influencers in these three pathways (Figure 4G). Collectively, these results suggest that both endocrine and non-endocrine cell clusters in the pituitary gland adapt to the physiological changes in the maternal body during pregnancy through elaborate regulatory mechanisms.

### 3.4. Characteristics of Various Subtypes of Gonadotroph in the Pituitary

**Figure 5:**
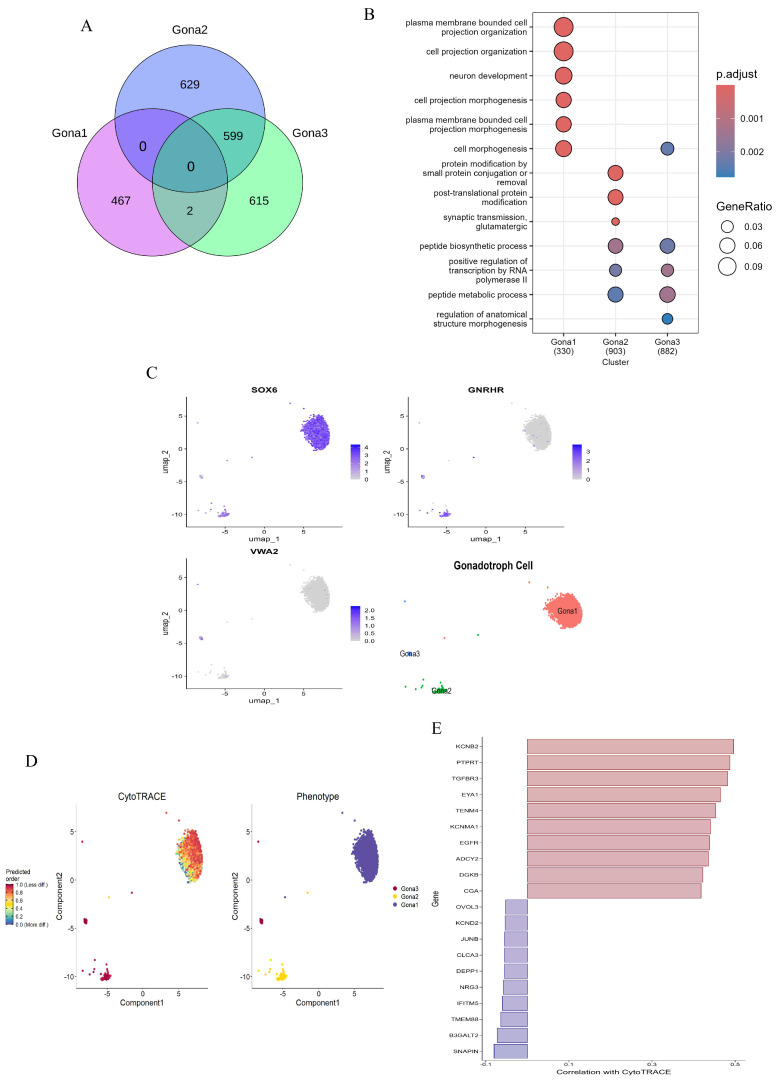
Molecular characterization of gonadotroph subtypes. (**A**): Venn diagram showing the intersection of DEGs between the three Gona subtypes and the amount of DEG unique to each subtype. (**B**): GO enrichment of DEGs of subtype gonadotrophs. (**C**): Marker genes of three subtypes and UMAP of dimensionality reduction. (**D**): The differentiation potential of gonadotropin cells was demonstrated by CytoTRACE analysis. The ability to differentiate from less to more is represented by a gradient color from red to blue. (**E**): Genes associated with differentiation potential are positively correlated in red and negatively correlated in blue.

Pituitary gonadotroph subtypes play a fundamental role in the specific response mechanisms to the pulsatile regulation of hypothalamic GnRH and the negative feedback of ovarian steroid hormones. In this study, a detailed exploration of the functions of gonadotroph subtypes was carried out. A total of 2115 gonadotrophs were meticulously classified into three subtypes (Gona1–Gona3). To elucidate the differences in gene expression and function among these subtypes, 467, 628, and 615 unique DEGs were identified in the Gona1, Gona2, and Gona3 clusters (Figure 5A,C), respectively. GO analysis of these DEGs revealed functional enrichments that were consistent with their respective gene expression patterns (Figure 5B). To assess the differentiation potential of these subtypes, CytoTRACE analysis was employed. The results indicated that Gona2 and Gona3 have a higher differentiation potential compared to Gona1, suggesting that Gona1 may possess more complex structures and functions (Figure 5D). Additionally, a series of positive and negative regulatory genes involved in gonadotroph differentiation were identified. These include *EYA1*, which is essential for pituitary lineage-specific differentiation, and *JUNB*, which promotes FSH secretion (Figure 5E). These findings demonstrate that the pituitary gland harbors multiple functionally distinct gonadotroph subtypes, each potentially playing unique roles in the overall regulation of the HPO axis.

### 3.5. Utilizing hdWGCNA to Identify Module Genes Associated with Pregnancy Maintenance in Luteal Cells

**Figure 6:**
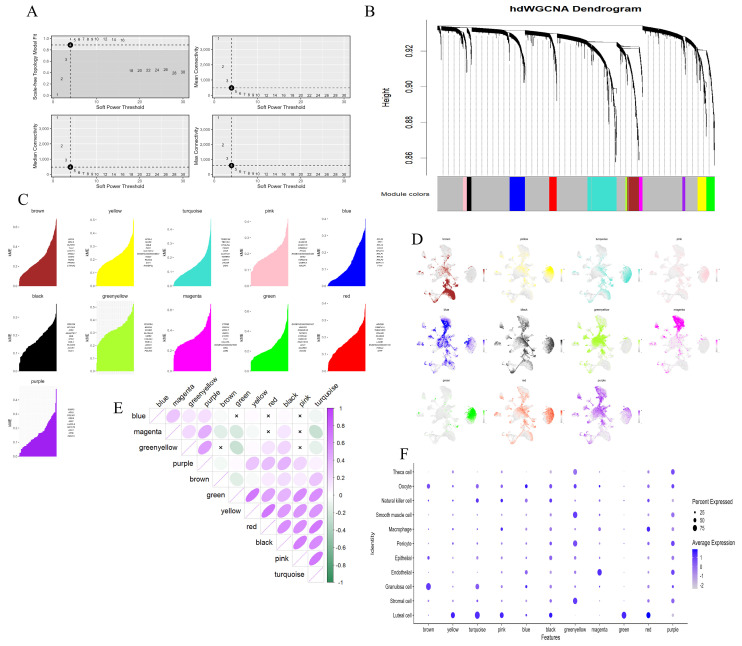
hdWGCNA identifying key genes involved in the maintenance of pregnancy by luteal cells. (**A**): The selection of the optimal soft threshold. (**B**): The coexpression network was constructed based on the ideal “4” soft threshold, and the genes were divided into several modules to obtain the gene clustering tree. The top half is the hierarchical clustering tree of genes and the bottom half is the gene module, which is the network module. (**C**): Feature-based gene connectivity was calculated for each gene in the coexpression network analysis to identify highly connected genes in each module. (**D**): The gene score of each module gene was calculated based on UCell algorithm. (**E**): Correlation between modules based on Pearson correlation analysis. (**F**): The expression of different cell types in each module is indicated by the size of the dots, with larger dots representing higher expression levels. The color intensity, ranging from light to dark, indicates the proportion of expression.

As the primary gland responsible for the secretion of P4 and E2 after ovulation, the corpus luteum is crucial for the maintenance of pregnancy function of the ovary [29,30]. In view of this, the present study specifically selected the luteal cell population as the research subject and conducted a hdWGCNA analysis. During the hdWGCNA analysis, we first carried out a power calculation; that is, we determined the soft threshold, to measure the degree of association between genes. Compared with the traditional hard threshold method, this approach can significantly enhance the strong correlations between genes, making the correlation values more in line with the characteristics of a scale-free network and thus having higher biological research value. After rigorous calculations and analyses, the optimal value of the soft threshold in this study was determined to be 4, which effectively ensured an appropriate balance between the sparsity and connectivity of the network (Figure 6A). Subsequently, we further calculated the topological overlap matrix (TOM), which is used to precisely represent the similarity of gene expression. Specifically, if two genes have similar expression patterns, they will exhibit a higher similarity value in the TOM matrix. The clustering tree generated by WGCNA visually demonstrated the clustering of genes, and during the clustering process, genes with stronger correlations were reasonably grouped into the same branch. Through in-depth analysis of this clustering tree, we successfully identified 11 modules, which were represented by different colored blocks in the tree (Figure 6B). Each module contains a group of genes with similar expression patterns (Figure 6C) and the gray module represents genes that do not belong to any of the identified modules. To gain a more in-depth understanding of the characteristic distribution of each module in the cell clusters, we calculated the module eigengene (ME) and identified highly correlated genes within each module, namely harmony module eigengenes (hME) (Figure 6D). On this basis, we further calculated the correlations between modules and finally determined the correlations between each module and various cell types in the ovary (Figure 6E,F). Based on the above analysis results, we found that the luteal cells showed the strongest correlations with six modules: yellow, turquoise, pink, black, green, and red. Based on this finding, we performed GO and KEGG functional enrichment analyses on the top 10 genes in these six modules that had the strongest correlations with the luteal cells. The analysis results indicated that the functions of these key genes cover multiple biological functions that directly support pregnancy maintenance, such as hormone-mediated signaling pathways, angiogenesis, blood vessel development, and calcium ion transmembrane transport, and also involve key pathways for successful pregnancy, such as steroid biosynthesis, calcium signaling pathways, renin secretion, and vascular smooth muscle contraction (Supplementary Figure S4A,B). In addition, the study also found that these modules also have strong correlations with other cell types in the ovary.

### 3.6. Cell–Cell Communication Among Various Cell Types in the Ovary

**Figure 7:**
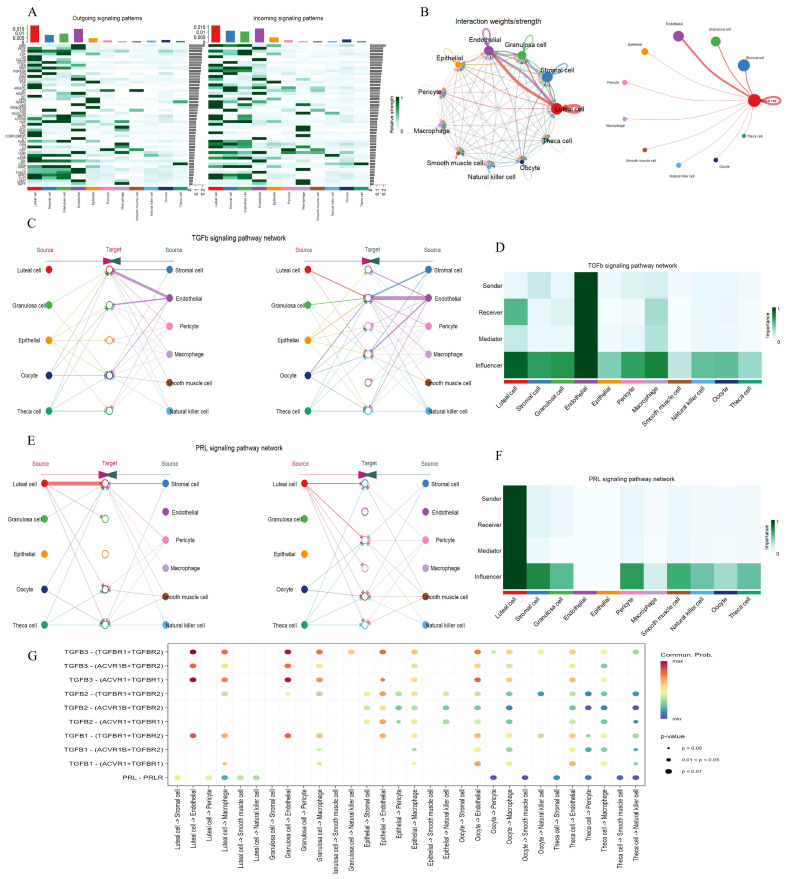
Signaling pathway networks and cell communication of various cell types within the ovary. (**A**): Identified signaling pathways between all cell types. (**B**): Circle plot illustrating the strength of interaction between the various cell types. (**C**,**E**): Hierarchical plots showing the intercellular communication network of TGFβ and PRL signaling pathways. (**D**,**F**): Heatmap showing the relative importance of cell type in TGFβ and PRL signaling pathways. (**G**): Bubble plot showing the strength of the interaction of ligand–receptor pairs in cellular communication in the TGFβ and PRL signaling pathways.

Based on the hdWGCNA analysis, gene modules associated with luteal cells not only participate in steroid hormone synthesis and vascular remodeling but may also mediate inter-cellular signal transmission by encoding ligand or receptor molecules. To systematically decipher this mechanism, this study integrated ovarian snRNA data to construct a communication network between luteal cells and microenvironment cells. In the ovaries of a pregnant cow, a total of 174 significant ligand–receptor pairs belonging to 50 signaling pathways were identified (Figure 7A). Given the functional characteristics of luteal cells, the research focused on the interaction mechanism between the TGFβ and PRL signaling pathways. Analysis of cell interaction intensity showed that the communication intensity between luteal cells and stromal cells, endothelial cells, and GCs was the most significant (Figure 7B). Network centrality analysis further revealed that endothelial cells function in a coordinated way to regulate the activity of the TGFβ pathway through autocrine and paracrine interactions with luteal cells (Figure 7C,D and Figure S4C). They highly express the receptor genes *TGFBR1*, *TGFBR2*, *ACVR1*, and *ACVR1B*, suggesting their function as core nodes for signal transmission (Figure 7G). Meanwhile, stromal cells and GCs mainly act as influencers in this pathway, indirectly enhancing pathway activity through epigenetic regulation or metabolic reprogramming (Figure 7C,D). In the PRL pathway, luteal cells exhibit strong autocrine characteristics, significantly expressing the prolactin ligand gene *PRL* (Figure 7E,G). Additionally, luteal cells further amplify signal output through paracrine interactions with stromal cells and pericytes, both of which highly express the prolactin receptor gene *PRLR* (Figure 7E–G and Figure S4D). This dual-action mode (autocrine and paracrine) may support pregnancy maintenance through the following mechanisms: luteal cells self-activate via the *PRL*–*PRLR* axis to regulate local progesterone synthesis and create an immune-tolerant microenvironment; stromal cells and pericytes receive signals through *PRLR* to promote angiogenesis and extracellular matrix remodeling. These findings reveal that luteal cells form a functional synergy with microenvironment cells through the TGFβ and PRL pathways to jointly maintain hormonal homeostasis and placental development during pregnancy.

### 3.7. GC Subtypes Developmental Trajectory and Cell–Cell Communication in the Ovary

**Figure 8:**
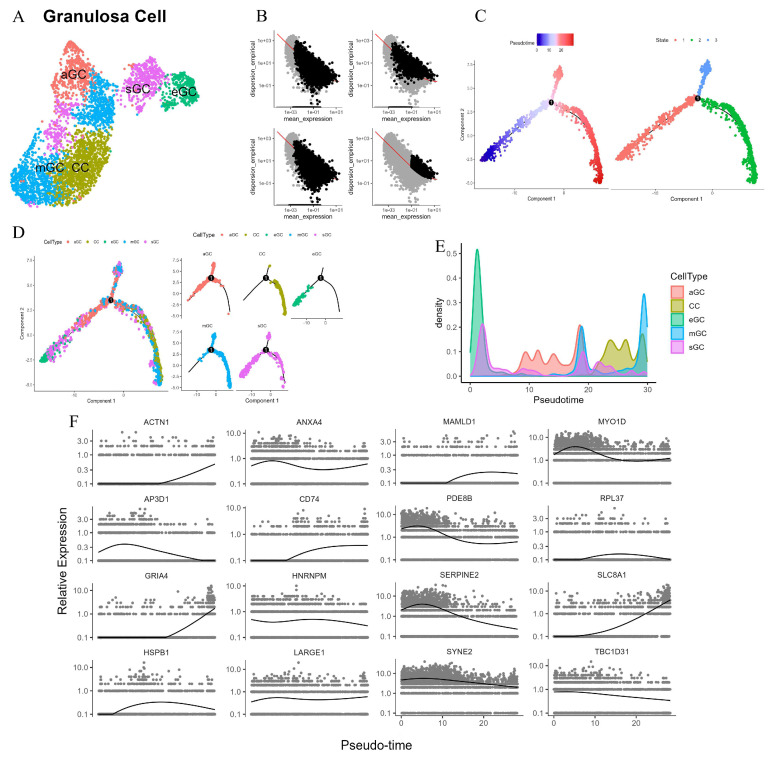
Identification of cow subtype granulosa cells in the ovary. (**A**): Granulosa cell subtype identification in UMAP. (**B**): Integrating multiple approaches to define cell progression trajectories, differentialGeneTest identifies significant genes (FDR < 0.01) in the top left panel, Seurat selects highly variable genes (HVGs) in the top right panel, cluster-specific differential expression patterns are resolved in the bottom left panel, and Monocle2 reconstructs pseudotemporal trajectories using HVGs in the bottom right panel. (**C**): Pseudotime trajectory analysis of GCs. (**D**): The subtypes of GCs distribution in pseudotime and number 1 represents the differentiation node. (**E**): The density distribution of each subtype GCs in pseudotime series. (**F**): The expression levels of the 16 genes with the highest degree of variation on the pseudo-temporal timeline.

During the physiological cycle of the female ovary, ovarian follicles undergo a series of complex and orderly developmental and maturation processes. Among these, luteinization, a crucial stage, exerts a profound influence on reproductive physiological functions [31]. After ovulation, the corpus luteum is formed by the somatic cells within the follicle, namely the theca cells and GCs, and plays a key role in maintaining pregnancy [31]. Specifically, the theca cells differentiate into small luteal cells, while the GCs undergo luteinization to produce large luteal cells [31,32]. The formation and effective maintenance of the corpus luteum’s function highly depend on the precise proliferation and differentiation processes of GCs and theca cells [31]. However, to date, the molecular mechanisms regulating luteinization in ovarian GCs have not been fully elucidated. Based on this, in our study, the GCs identified from the ovary were divided into seven clusters, labeled as C0–C6 (Figure 8A). Referring to the findings of other studies on ovarian GCs, these seven clusters were further classified into five subtypes. Specifically, C0 was identified as cumulus GCs (CC), characterized by *PAPSS2*^+^ and *FST*^+^; C1 and C2 were classified as mural GC (mGC), showing *INHBA*^+^; C3 was atretic GC (aGC), presenting *GJA1*^−^ and *CDH2*^−^; C4 and C6 were steroidogenic GCs (sGC), marked by *PDE10A*^+^; and C5 was early GC (eGC), featuring *WT1*^+^ and *VCAN*^−^.

To reasonably sort the cells, this study initially employed multiple methods for gene screening. These included selecting significant genes through differentialGeneTest, identifying highly variable genes using Seurat, choosing differentially expressed genes via Clusters, and picking highly variable genes with Monocle (Figure 8B). After a comprehensive evaluation of the effectiveness of these four methods, the third method was ultimately selected for cell sorting. Subsequently, by mapping the subtypes onto the pseudotemporal trajectory, it was discovered that there are three differentiation states in ovarian subtype GCs (Figure 8C). State 1 is in the early stage of pseudotime, while states 2 and 3 are in the late differentiation stage. GCs can differentiate into two main directions, namely mGC and CC. eGC is in the early stage of differentiation, and aGC exists in the early phases of states 1–3, which implies that aGC is the cell type that undergoes programmed cell death during the follicular development stage, before the follicle reaches the mGC stage. Notably, sGC persists throughout all states of the pseudotemporal process (Figure 8D). The results of cell density analysis and GO enrichment analysis further validate the above conclusions (Figure 8E and Figure S5A).

In addition to the pseudotemporal analysis, our study also conducted a cell–cell communication analysis on the five GC subtypes. The results showed that, in both the TGFβ signaling pathway and the PRL signaling pathway, the cell interaction between eGC and sGC was the strongest (Supplementary Figure S5B,G,H). They play an important regulatory role in these two signaling pathways through autocrine and paracrine mechanisms (Supplementary Figure S5C–F). Combining the results of the pseudotemporal analysis and the cell–cell communication analysis, it can be speculated that sGC may be similar to luteinized GCs, capable of providing the necessary hormonal environment for cows, thereby enabling the body to better adapt to the pregnant state.

### 3.8. Cell Type Deconvolution of the Spatially Resolved Cattle Ovary Transcriptome

**Figure 9:**
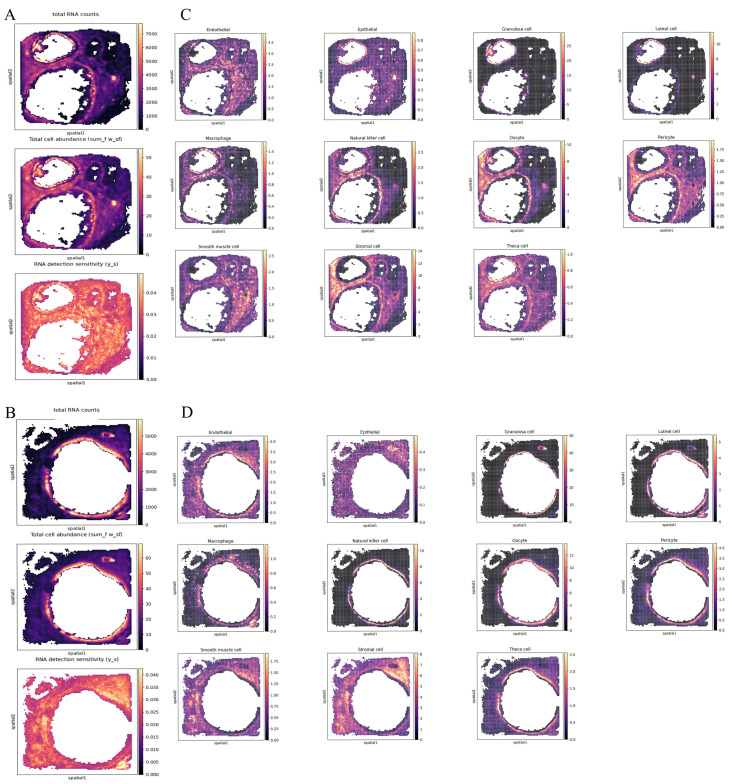
ST deconvolution of two ovaries from different angles based on snRNA-seq using Cell2location. (**A**,**B**): Spatial heatmap illustrating the number of total RNA counts, total cell abundance and RNA detection sensitivity of the ovary from two different angles. (**C**,**D**): Spatial heatmap showing the spatial localization based on the abundance of marker genes for the major ovarian cell types identified in snRNA-seq.

Although snRNA-seq has dissected the cellular heterogeneity within ovarian tissue, the spatial positions of cells are lost during tissue dissociation. Therefore, to better examine the spatial positions of the major cell types in the ovaries of pregnant Mongolian cows, we conducted Stereo-seq analysis on two different angles of the right ovary. Using the snRNA-seq data as a reference, we performed cell type deconvolution on the ST data to carry out fine localization analysis with the Cell2location algorithm. Cell type deconvolution enables high-resolution localization of reference cell types in a spatial context, offering an unparalleled opportunity to explore gene expression profiles in situ [33,34]. The spatial heatmaps show that there are follicles of various sizes in the ovaries from the two different angles. It can be observed that cells are relatively dense around the follicular membrane, and the RNA capture efficiency is the highest there, suggesting it might be a functionally active area (Figure 9A,B). In contrast, the peripheral fibrotic tissue of the ovary exhibits low cell abundance but also has a high RNA capture rate, indicating that this area may consist of quiescent cell populations. Based on the spatial deconvolution analysis using Cell2location, we successfully resolved the 11 major cell types previously identified in the snRNA-seq within the ovarian tissue sections (Figure 9C,D). The relative abundances of each cell type were mapped to the spatial coordinates through color gradients or dot densities, revealing the cellular compositional heterogeneity in the functional regions of the ovary. GCs are concentrated around the follicular antrum, forming a ring-like structure that highly overlaps with the position of the oocyte. Luteal cells aggregate in discrete patches and are located in the ovarian stromal region, co-localizing with endothelial cells and pericytes. Macrophages and natural killer cells are distributed around the corpus luteum. Endothelial cells, pericytes, and smooth muscle cells form a continuous network that traverses the follicular and luteal regions. Theca cells are mainly enriched in the peripheral area of the follicle, adjacent to the area with a high abundance of GCs, forming a double-layer structure with GCs in the inner layer and theca cells in the outer layer. Stromal cells are widely distributed in the ovarian stromal region, forming a continuous matrix network that runs through the follicular, luteal, and vascular regions.

## 4. Discussion

In this study, we obtained a total of 6161 high-quality nuclei from the hypothalamus, 14,715 from the pituitary, and 26,072 from the ovary, allowing for robust and high-resolution single-nucleus transcriptomic profiling across the entire HPO axis. These datasets enabled the identification of diverse cell populations and detailed intercellular communication networks underlying reproductive regulation during pregnancy in Mongolian cattle.

The hypothalamus is a central hub organ that integrates neuroendocrine signals and plays a crucial role in reproductive regulation. Using the snRNA-seq technology, we identified 15 transcriptionally heterogeneous cell clusters in the hypothalamus of Mongolian cattle. Among them, neurons account for 53% of the total cell population. Neurons showed prominent expression of *CACNA1E*, whose functions were enriched in synaptic signaling and neuroendocrine regulation in this study, indicating its central role in neural signal integration during pregnancy. Non-neuronal cell populations maintain microenvironmental homeostasis through neuron–glia interactions [35]. By subdividing glutamatergic (Glut1–Glut7) and GABA1 subtypes based on the expression of GLS and GABBR2, we discovered a hierarchical differentiation trajectory. Glut4 emerged as a key regulatory hub in TGFβ signaling, expressing *TGFBR1* and *ACVR1* receptors related to hypothalamic–pituitary negative feedback, which may regulate pituitary hormone secretion through negative feedback. Glial cells regulate the activity of GnRH neurons through TGFβ, promoting the release of GnRH hormones [36,37]. The PRL signaling pathway is mainly dominated by GABA1, which shows strong expression of PRL ligands, potentially reflecting the adaptive changes in maternal behavior and lactation in cows during pregnancy [38]. Notably, there was a lack of intrinsic FSH signaling in the neuronal cell clusters, suggesting that the hypothalamic regulation of FSH depends on input signals from the pituitary [39]. Trajectory analysis indicated that Glut4 represents an early neuronal state that differentiates into GABA1, marked by the dynamic expression of the synaptic adhesion molecule *NLGN1*, which is crucial for the maturation of neural circuits [40]. Meanwhile, the biphasic expression of *ENSBTAG00000049814* and the decline in *ABCC13* expression suggest the existence of stage-specific regulatory programs during neuronal maturation.

Our research has discovered a complex regulatory network between endocrine and non-endocrine cells in the pituitary gland of pregnant cows. Pituitary stem cells account for 32% of the total cell population. Their high-frequency cell–cell interactions suggest that they dynamically replenish the endocrine cell population through the TGFβ pathway. This finding is consistent with the pluripotent characteristics of stem cells, which can differentiate into all endocrine cell types in the embryonic and adult pituitary [41]. Notably, somatotrophs exhibit strong autocrine ability in the TGFβ pathway. This may be through a local signal amplification mechanism mediated by the *TGFBR1/ACVR1* receptors, collaborating with the hypothalamic GHRH signal to maintain growth hormone homeostasis [42]. Additionally, somatotrophs may be involved in inhibiting the excessive secretion of gonadotropins induced by GnRH pulses. Gonadotrophs form a dual-regulatory mode through TGFβ autocrine and FSH paracrine [42]. This mode may regulate the pulsatile secretion of gonadotropins by activating the inhibin–activin system, which corresponds to the regulatory requirements of fluctuating gonadotropin levels on ovarian luteal function during pregnancy. Lactotrophs, as the core hub of the PRL pathway, form a *PRL–PRLR* paracrine network with stem cells. This network may be driven by the increased E2 levels during pregnancy, synergistically promoting mammary gland development [43]. Non-endocrine cells, such as macrophages, provide immune microenvironment regulation for endocrine cells [44]. In particular, the gonadotroph subtypes (Gona1–Gona3) show hierarchical functional differentiation. The high differentiation potential of Gona2/Gona3 is associated with enhanced FSH secretion mediated by *JUNB*, while the complex functional characteristics of Gona1 may correspond to the specific response to the negative feedback of ovarian steroid hormones [45]. The study also found that pituitary stem cells dominate the FSH pathway through the *CGA* ligand, suggesting that they may directly regulate gonadotropin synthesis, rather than the traditional view of only indirectly regulating through hypothalamic GnRH. This breakthrough discovery provides a new perspective for understanding the autonomous regulation of the HPO axis during pregnancy. The current results, together with the previous hypothalamic research (Glut4-TGFβ hub and GABA1–PRL axis), outline a bidirectional neuroendocrine regulatory network. The hypothalamus inhibits excessive pituitary activation through TGFβ signals, while pituitary stem cells integrate ovarian feedback through FSH signals, forming a pregnancy-specific homeostasis maintenance mechanism. Subsequent research needs to combine spatio-temporal multi-omics technologies to analyze the three-dimensional interaction patterns of stem cells in the pituitary microenvironment and their dynamic response characteristics during the perinatal hormonal storm.

hdWGCNA analysis of ovarian luteal cells revealed that genes in the green module were enriched in steroid synthesis-related pathways. *HMGCR* and *HMGCS1* are the core enzymes of the mevalonate pathway, responsible for converting acetyl-CoA to mevalonate, providing precursors for cholesterol and steroid hormone synthesis. Studies have shown that LH enhances the P4 synthesis ability of luteal cells by up-regulating their expression [46]. *CYP51A1*, also known as lanosterol 14α-demethylase, catalyzes the oxidation reaction in cholesterol biosynthesis. Its expression level directly affects the homeostasis of the cholesterol pool in luteal cells. This gene has been identified as a hub gene in the steroidogenesis-related modules in multiple studies [47]. *HDL* is the main pathway for luteal cells to obtain exogenous cholesterol. Its expression level is positively correlated with the efficiency of P4 synthesis [48]. Furthermore, *ACLY* and *ACSS2* regulate the production of acetyl-CoA, integrating glucose and lipid metabolism to provide substrates for cholesterol synthesis. RNA-seq data showed that these genes are highly expressed in luteal cells and are also modulated by the LH signal [46]. In addition, *LHCGR*, a characteristic gene of luteal cell, was identified in the turquoise module. During early pregnancy, hCG “rescues” the corpus luteum through *LHCGR*, inhibiting apoptotic signals and extending the lifespan of the corpus luteum until the 22nd week of pregnancy [49]. The functional integrity of *LHCGR* is crucial for pregnancy maintenance. Compound heterozygous mutations can lead to abnormal receptor folding or membrane-localization defects, causing Leydig cell hypoplasia or female luteal insufficiency, manifested as amenorrhea or infertility [50,51]. Clinical data have shown that patients with inactivating mutations in *LHCGR* have no response to LH/hCG in luteal cells and need to rely on exogenous P4 to maintain pregnancy [51]. Moreover, the decrease in *LHCGR* expression is closely related to luteal regression: in the late luteal phase, despite the continuous presence of LH pulses, the reduced abundance of *LHCGR* makes luteal cells lose hormone responsiveness, ultimately leading to the termination of progesterone secretion [52,53]. The expression level of *LHCGR* in luteal cells is regulated by the GnRH pulse pattern. High-frequency GnRH pulses reduce LH secretion by down-regulating GnRHR, while low-frequency pulses enhance LH release, indirectly affecting *LHCGR* activity [52]. There is a close functional synergistic mechanism between luteal cells and the microenvironment, involving a coordinated regulatory network of the TGFβ and PRL pathways and multi-cell functional modules in the luteal microenvironment. In the regulation with endothelial cells as the core node of the TGFβ signal, TGFβ promotes angiogenesis by activating the SMAD1/5 pathway to meet the high blood supply requirements of the corpus luteum. On the other hand, it inhibits the differentiation of Th1/Th17 cells, promotes the function of Treg cells to maintain local immune tolerance, and regulates pericyte differentiation to maintain the integrity of the vascular basement membrane [54,55,56]. The PRL pathway has an autocrine–paracrine synergistic effect. In autocrine, the *PRL–PRLR* of luteal cells activates the JAK2/STAT5 pathway to increase P4 production [57]. In paracrine, PRL secreted by stromal cells causes pericytes to release VEGF to support the nutrient supply of luteal cells [58]. Meanwhile, PRL can also perform metabolic reprogramming through the PI3K/Akt/mTORC1 pathway and inhibit apoptotic signals to extend the survival of the corpus luteum [59]. In the multi-cell functional module of the luteal microenvironment, endothelial cells play a central role. They secrete factors to promote angiogenesis, regulate permeability to ensure hormone transport, and convert mechanical forces into biochemical signals to coordinate the development and regression of the corpus luteum [54,58,60]. There is a paracrine interaction between granulosa cells and luteal cells. *AMH* derived from GCs inhibits premature luteal regression. *PRL* activates *STAT3* in granulosa cells to promote the expression of aromatase, synergistically achieving the balance of E2 and P4 [61,62].

During the process of follicle development and atresia, there is a dynamic equilibrium among GC subtypes. sGCs, as pre-luteinized precursor cells, persist throughout the follicle development trajectory according to pseudotime trajectory analysis. They participate in luteinization by playing a central role in steroid synthesis. The highly expressed *PDE10A* gene encodes a dual-substrate phosphodiesterase that degrades cAMP and cGMP to regulate the activity of steroid-synthesizing enzymes [63]. Inhibition of *PDE10A* can increase cAMP levels, activate PKA, and promote the expression of steroid-generating genes [64]. Moreover, the high expression of *PDE10A* is of great significance for maintaining the local cAMP signaling homeostasis during pregnancy and ensuring the continuous secretion of progesterone during luteinization [65]. aGCs are in the early stage of the pseudotime trajectory. The down-regulation of their connexins, along with the absence of *GJA1* and *CDH2* expression, leads to the interruption of intercellular communication and the disintegration of the follicle structure [66]. Additionally, the apoptosis pathway is activated, and the imbalance of the Bcl-2 family protein ratio triggers apoptosis [67]. There are two viewpoints regarding function of aGCs. One is the active clearance mechanism, where aGCs are cleared through the death receptor and mitochondrial pathways, and the crosstalk between autophagy and apoptosis accelerates this process [66]. The other is the residual function hypothesis, which suggests that some aGCs may secrete factors such as *TGFβ* and *AMH* to inhibit the development of adjacent follicles and coordinate the dynamic balance of the ovarian follicle pool [67].

During follicle development, GCs undergo dynamic spatial reorganization, and their circular distribution around the follicular antrum is closely associated with the interaction between oocytes and somatic cells. In the primordial follicle stage, a single layer of flattened GCs envelops the primary oocyte, forming the zona pellucida and basement membrane [68,69]. As the follicle develops to the pre-antral stage, GCs differentiate into outer cuboidal cells and inner columnar cells, and an antrum is formed [69]. This stratified structure not only provides physical support for the oocyte but also mediates bidirectional signal transmission through gap junctions and extracellular vesicles [70]. For example, the oocyte regulates the proliferation and differentiation of GCs by secreting factors such as *BMP15* and *GDF9*, while the E2 and anti-Mullerian hormone produced by GCs feedback-regulate oocyte maturation [70]. Moreover, the interaction between GCs and theca cells promotes angiogenesis through VEGF and bFGF, providing nutrients for the follicle [71]. Thus, the circular distribution of GCs is not only a structural requirement but also an embodiment of functional collaboration, with their spatial positioning directly supporting oocyte development and the homeostasis of the follicular microenvironment. During corpus luteum formation, the co-localization of luteal cells with endothelial cells and pericytes is crucial for the vascularized structure of the corpus luteum. After ovulation, the basement membrane of the original follicle ruptures, and endothelial cells invade the GCs layer from the theca layer, forming a dense vascular network [71]. VEGF-A, secreted by luteal cells, drives the proliferation and migration of endothelial cells [72,73]. Co-culture experiments show that endothelial cells form island-like structures in the early stage, with pericytes distributed around their tips, and gradually form a network-like vasculature in the later stage [72]. Macrophages exhibit dynamic distribution during the corpus luteum cycle, with relatively low numbers in the early luteal phase and significant enrichment in the late luteal regression phase [74]. In early pregnancy, hCG inhibits macrophage infiltration by down-regulating *MCP-1*, protecting the corpus luteum from immune attack [74]. Additionally, macrophages may inhibit T-cell activation by secreting *IL-10* or *TGFβ*, maintaining the immune-tolerant state of the corpus luteum [75]. Although there is limited research on NK cells in the normal corpus luteum, studies related to ovarian cancer suggest that they may be involved in microenvironmental regulation. In the tumor microenvironment, NK cells have impaired function due to the down-regulation of activating receptors [76]. A similar mechanism may limit the attack of NK cells on healthy luteal cells in the corpus luteum. NK cells may also participate in maintaining the immune homeostasis of the corpus luteum by secreting IFN-γ or directly contacting cells to suppress excessive inflammatory responses [76]. However, their specific roles in corpus luteum regression or pregnancy maintenance still need further verification.

Although this study focused on pregnant Mongolian cattle, many of the regulatory features uncovered within the HPO axis appear to be conserved across cattle breeds and mammalian species. For instance, the activation of TGFβ signaling in luteal and granulosa cells is consistent with prior findings in other species, where this pathway is known to modulate steroidogenesis, angiogenesis, and immune tolerance within the ovary [77]. The *PRL–PRLR* autocrine–paracrine loop, prominently detected in both hypothalamic and ovarian compartments, mirrors established roles for prolactin in luteal maintenance and mammary development reported in goat [78]. Moreover, the presence of stem-like cells in the pituitary and their association with endocrine lineages via TGFβ signaling aligns with studies of fetal pituitary development in humans, where plasticity is critical for hormonal adaptability [79]. The molecular markers and pathways highlighted here may inform the development of diagnostic tools or reproductive interventions aimed at improving fertility, especially under harsh environmental conditions where reproductive resilience is crucial.

Despite providing novel insights into the cellular architecture and inter-organ signaling of the HPO axis in pregnant Mongolian cattle, this study has several limitations that warrant consideration. First of all, while snRNA-seq enables transcriptomic profiling from frozen tissues and allows for the capture of cell types that are otherwise difficult to isolate, it lacks information on cytoplasmic transcripts and cellular morphology, which may limit cell type resolution and downstream interpretation. In addition, the ST offers only spot-level resolution, and cannot distinguish single-cell features within mixed-cell regions. As such, cell localization was inferred based on marker gene distribution and histological context, but not directly confirmed at the single-cell level. Moreover, due to the complexity and heterogeneity of HPO tissues, certain known cell types—such as specific hypothalamic neuroendocrine subtypes, pituitary hormone-secreting cells, or rare ovarian stromal components—may have escaped detection or lacked sufficient transcriptomic depth for confident annotation. The inferred cell–cell interactions and signaling networks are based on computational predictions and, although biologically plausible, they require further experimental validation. Likewise, the biological relevance of enriched signaling pathways and gene modules remains to be confirmed via functional assays. The specificity of some marker genes also requires cross-species comparison to validate their conservation and reproducibility across mammalian models. In addition, although efforts were made to control biological variability by sampling from a single pregnant cow, unmeasured confounding variables—including individual physiological state, environmental stress, or nutritional status—may still influence gene expression profiles. Finally, while this study outlines a cellular framework of the pregnancy-stage HPO axis, the proposed signaling mechanisms remain speculative. Future studies should focus on validating these findings through targeted in vivo and in vitro experiments, expanding to additional individuals and developmental stages, and integrating multi-omics approaches to examine the functional hierarchy of gene regulation during reproduction.

## 5. Conclusions

Our study of the HPO axis in pregnant Mongolian cattle provides a descriptive framework that suggests potential regulatory mechanisms involved in reproductive maintenance. The hypothalamus appears to engage Glut4-TGFβ and GABA1-PRL signaling pathways that may contribute to pituitary feedback modulation, while pituitary stem cells are implicated in maintaining endocrine plasticity through TGFβ signaling, complementing and potentially expanding current models of GnRH-mediated regulation. In the ovary, luteal cells likely participate in steroidogenesis and immune modulation via endothelial-centered TGFβ networks and PRL-associated autocrine–paracrine interactions, supported by granulosa–theca communication. The spatial organization of granulosa cells reveals positional and transcriptional heterogeneity, with a subset (e.g., PDE10A-positive GCs) potentially serving as luteal precursors. While these findings are based on transcriptomic and spatial data, experimental validation will be essential to confirm these proposed mechanisms. Future studies should integrate spatial-temporal multi-omics and functional assays to further explore 3D cell–cell interactions and assess candidate pathways for therapeutic targeting in luteal dysfunction and ovarian disorders. Collectively, this work offers a foundational atlas that may inform future investigations into reproductive adaptation and fertility optimization in livestock.

## Data Availability

The datasets presented in this article are not readily available because the data are part of an ongoing study.

## Supplementary Materials

The following supporting information can be downloaded at: https://www.mdpi.com/article/10.3390/ani15152277/s1, Figure S1: Raw sequencing data and quality control plots; Figure S2: Expression characteristics of marker genes for each cell type in all HPO axis tissues; Figure S3: Molecular characterization of subtype neurons in the hypothalamus; Figure S4: Molecular characterization of granulosa cells in the ovary; Figure S5: Molecular characterization of granulosa cells in the ovary.

## Abbreviations

The following abbreviations are used in this manuscript:

HPO	Hypothalamus–pituitary–ovarian
snRNA-seq	Single-nucleus RNA sequencing
ST	Spatial transcriptomics
P4	Progesterone
GnRH	Gonadotropin-releasing hormone
FSH	Follicle-stimulating hormone
LH	Luteinizing hormone
scRNA-seq	Single-cell RNA sequencing
Gona	Gonadotrophs
GHRH	Growth hormone-releasing hormone
CRH	Corticotropin-releasing hormone
TRH	Thyrotropin-releasing hormone
GCs	Granulosa cells
LB	Lysis Buffer
NB	Nuclei Buffer
NB-RNA	Nuclei Buffer with RNA protection
BSA	Bovine serum albumin
GEMs	Gel beads-in-emulsions
UMIs	Unique molecular identifiers
DEGs	Differentially expressed genes
GO	Gene Ontology
KEGG	Kyoto Encyclopedia of Genes and Genomes
hdWGCNA	High-dimensional weighted gene co-expression network analysis
MEs	Module eigengenes
H&E	Hematoxylin–eosin
E2	Estrogen
TOM	Topological overlap matrix
ME	Module eigengene
hME	Harmony module eigengenes
NMF	Non-negative matrix factorization
