# Supplementary material for: Single-Nucleus RNA Sequencing and Spatial Transcriptomics Reveal Cellular Heterogeneity and Intercellular Communication Networks in the Hypothalamus–Pituitary–Ovarian Axis of Pregnant Mongolian Cattle
Source: Animals (Basel). 2025 Aug 4;15(15):2277. doi: 10.3390/ani15152277 (PMC12345466; doi:10.3390/ani15152277)
Supplement: Supplementary file 1 [file animals-15-02277-s001.zip › animals-3702025-supplementary.pdf]

A

Sample ID	Number of cells	Total Genes	Mean Reads per Cell	Median UMI per Cell	Median Genes per Cell	Mapping rate(%)	Q30(%)
**HT**	**6,664**	**19,634**	**50,417**	**5,010**	**2,066**	**93.6**	**93.7**
**PG**	**14,830**	**20,410**	**26,730**	**3,314**	**1,910**	**93.4**	**94.0**
**CL**	**12,078**	**19,869**	**31,742**	**2,767**	**1,592**	**93.8**	**93.7**
**DF**	**3,787**	**19,548**	**91,092**	**1,825**	**1,207**	**89.1**	**93.6**
**FL**	**1,251**	**16,168**	**36,439**	**1,500**	**956**	**79.1**	**93.9**
**GF**	**9,036**	**19,817**	**40,712**	**1,544**	**1038**	**93.7**	**93.5**

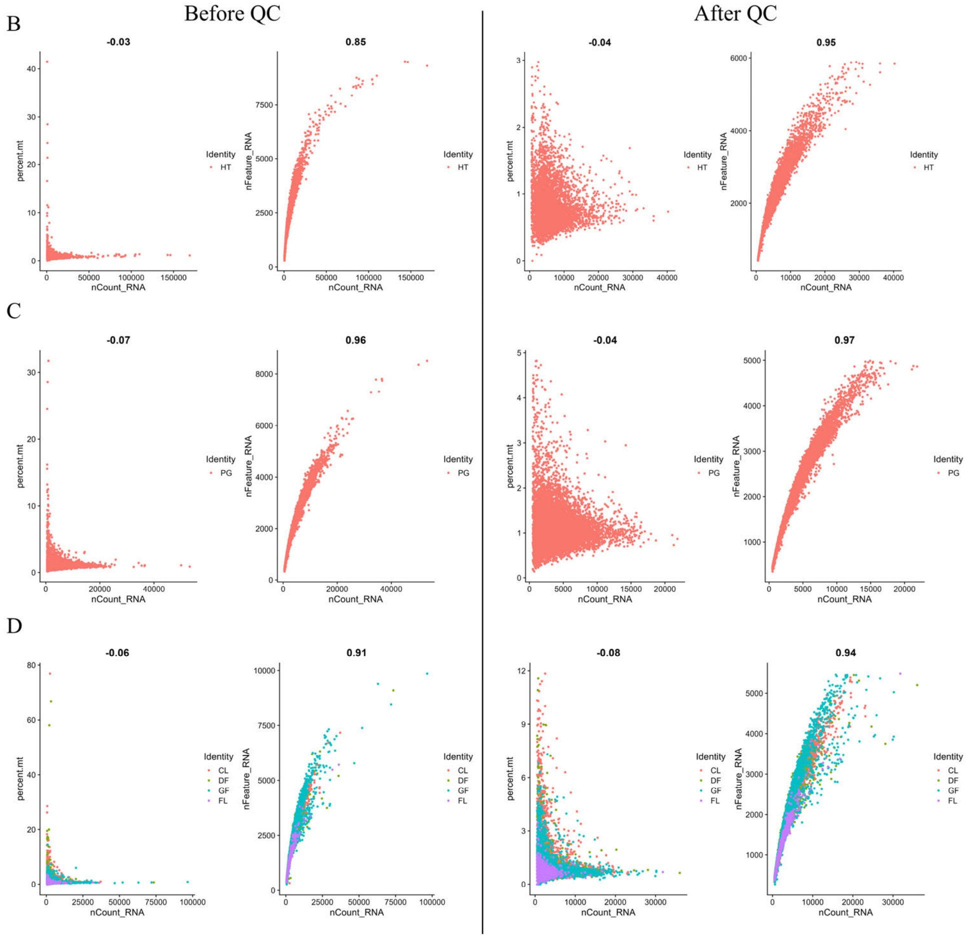

Supplementary Figure S1. Raw sequencing data and quality control plots.

A: Raw sequencing data. Scatterplot showing the quality control of single-nucleus transcriptome data in the hypothalamus (B), pituitary (C), and ovary (D).

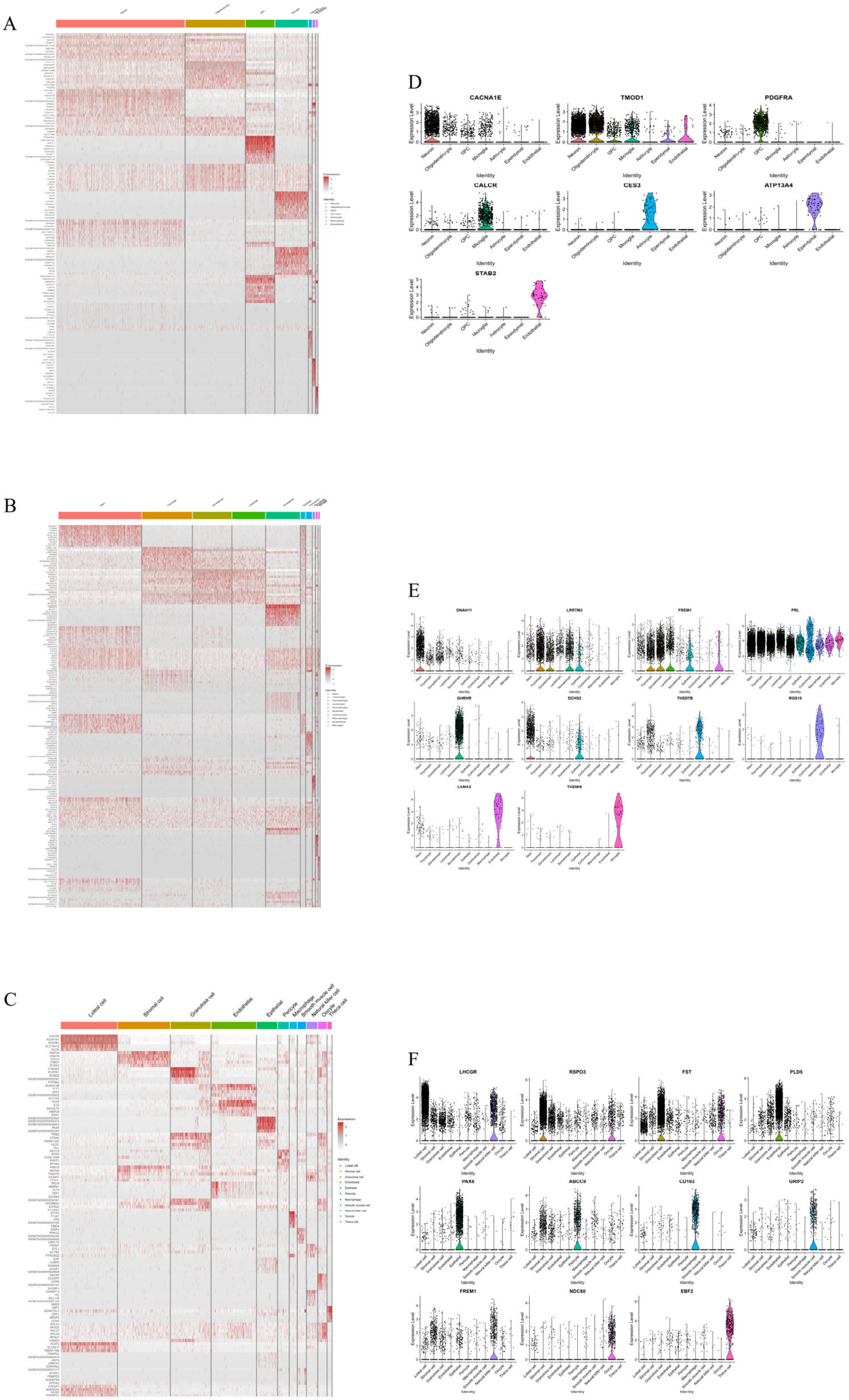

Supplementary Figure S2. Expression characteristics of marker genes for each cell type in all H-P-O axis tissues.

A-C: Heatmap showing the top 10 genes in each cell type in the hypothalamus, pituitary, and ovary. D-E: Violin plots visualizing expression specificity of the marker genes for each cell type in the hypothalamus, pituitary, and ovary.

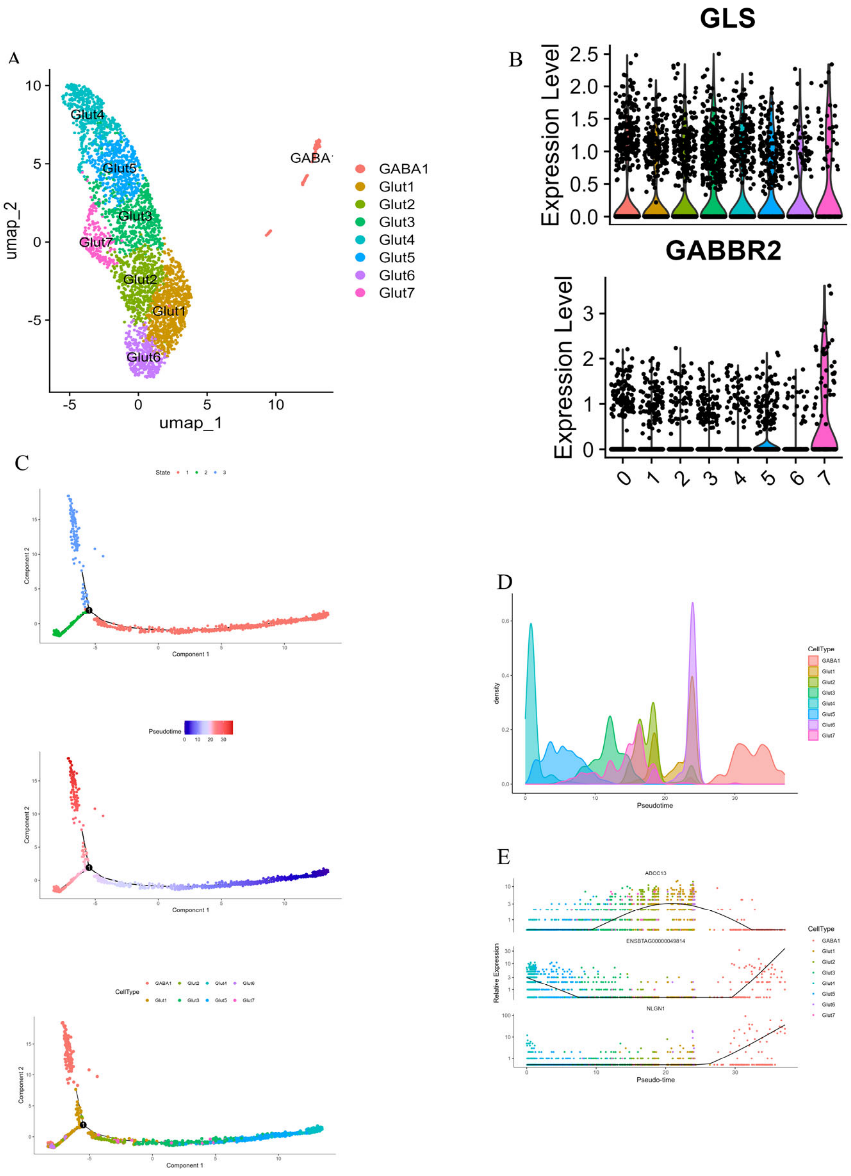

Supplementary Figure S3. Molecular characterization of subtype neurons in the hypothalamus.

A: UMAP clusters 8 neuronal subtypes in the hypothalamus. B: Expression of *GLS* and

GABBR2 genes in each subcluster. C: Pseudotime analysis of neuronal subtypes. D: Density of neuronal subtypes in pseudotime analysis. E: Trends of genes with the highest variation in pseudotime timeline.

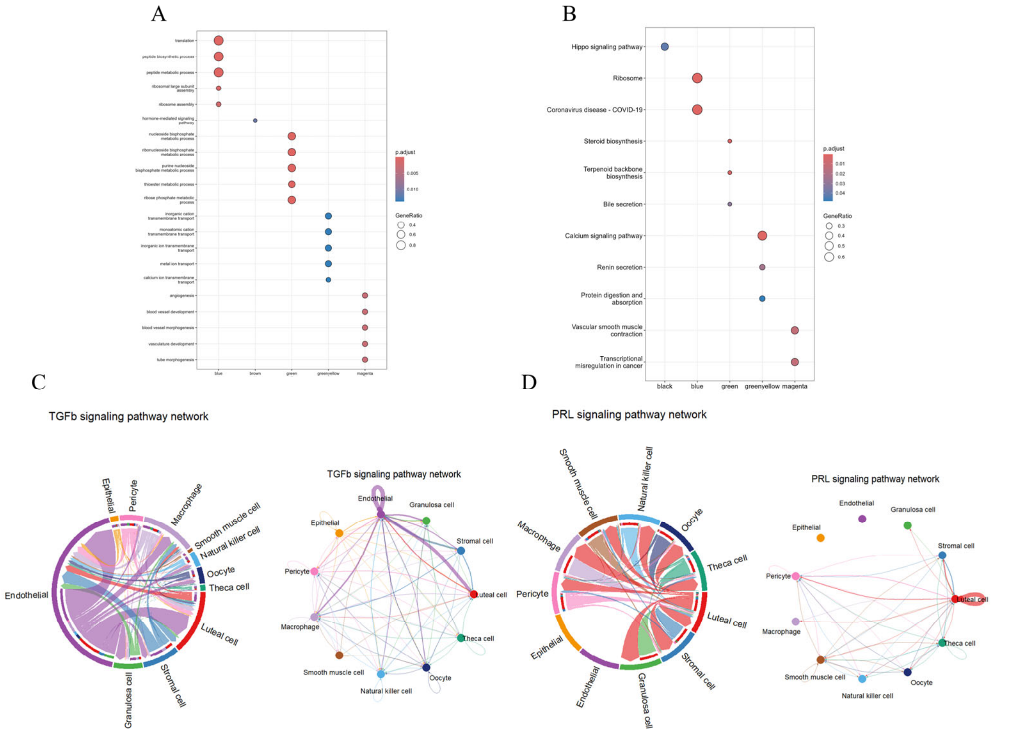

Supplementary Figure S4. Molecular characterization of granulosa cell in ovary.

A and B: GO and KEGG enrichment analysis of granulosa cell. B: Expression of *GLS* and *GABBR2* genes in each subcluster. C: Interaction of ovarian cell types in TGFβ signaling pathway. D: Interaction of ovarian cell types in PRL signaling pathway.

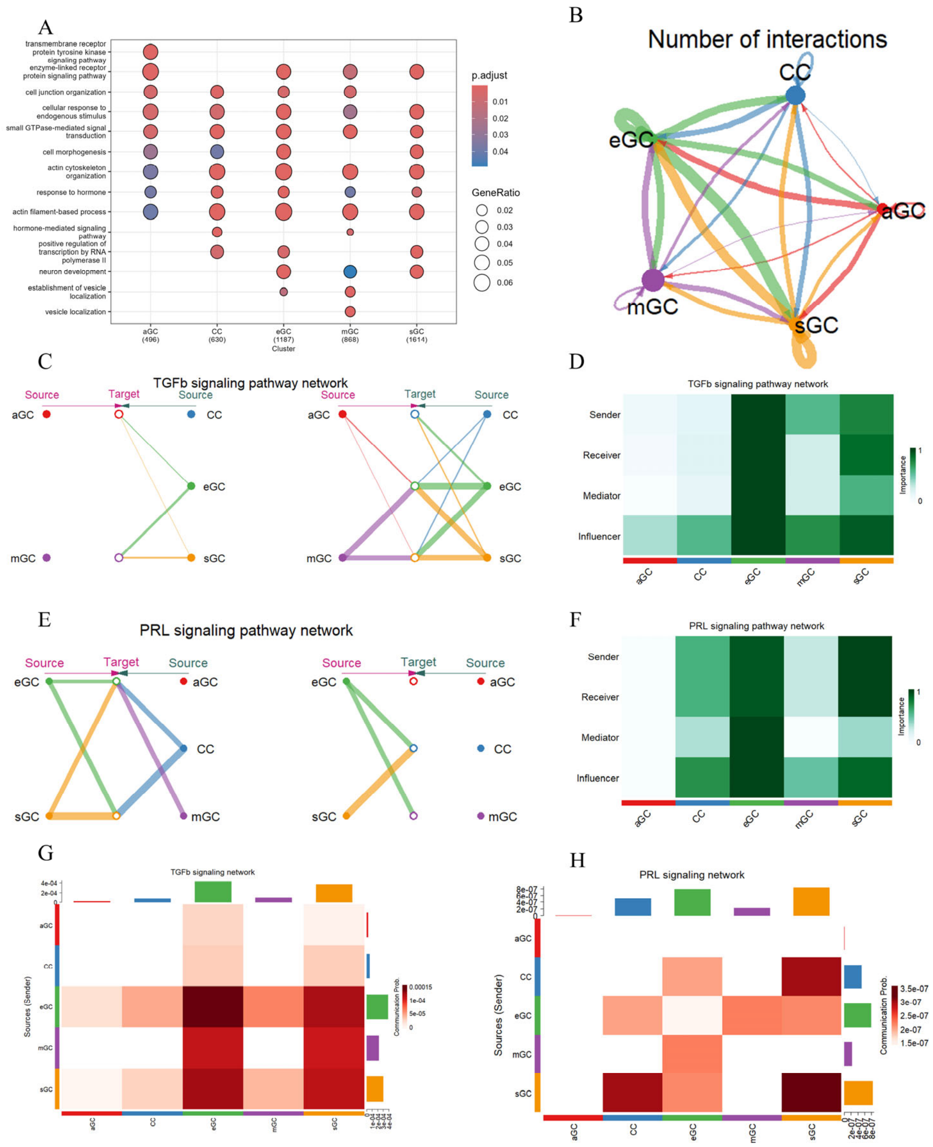

Supplementary Figure S5. Molecular characterization of granulosa cell in ovary.

A: GO enrichment analysis of differential genes in different subtypes of granulosa cell. B: Circle plot illustrates the interaction of the subtypes. C and E: Hierarchical plots show the intercellular communication network of TGFβ and PRL signaling pathways. D and F: Heatmap shows the relative importance of cell type in TGFβ and PRL signaling pathways. G and H: Heatmap shows the intensity of cell communication in TGFβ and PRL signaling pathways for each subtype.
